# Alpha-synuclein prevents the formation of spherical mitochondria and apoptosis under oxidative stress

**DOI:** 10.1038/srep42942

**Published:** 2017-02-22

**Authors:** Stefanie Menges, Georgia Minakaki, Patrick M. Schaefer, Holger Meixner, Iryna Prots, Ursula Schlötzer-Schrehardt, Kristina Friedland, Beate Winner, Tiago F. Outeiro, Konstanze F. Winklhofer, Christine A. F. von Arnim, Wei Xiang, Jürgen Winkler, Jochen Klucken

**Affiliations:** 1 Department of Molecular Neurology, University Hospital Erlangen, Friedrich-Alexander-University (FAU) Erlangen-Nürnberg, 91054 Erlangen, Germany; 2 Department of Neurology, Ulm University, 89081 Ulm, Germany; 3 IZKF Junior Research Group III and BMBF Research Group Neuroscience, IZKF, FAU Erlangen-Nürnberg, 91054 Erlangen, Germany; 4 Department of Stem Cell Biology, Institute of Human Genetics, FAU Erlangen-Nürnberg, 91054 Erlangen, Germany; 5 Department of Ophthalmology, University Hospital Erlangen, FAU Erlangen-Nürnberg, 91054 Erlangen, Germany; 6 Molecular and Clinical Pharmacy, Department of Chemistry and Pharmacy, FAU Erlangen-Nürnberg, 91054 Erlangen, Germany; 7 Department of Neurodegeneration and Restorative Research, University Medical Center Göttingen, 37073 Göttingen, Germany; 8 Max Planck Institute for Experimental Medicine, 37075 Göttingen, Germany; 9 Molecular Cell Biology, Institute of Biochemistry and Pathobiochemistry, Ruhr-University Bochum, 44801 Bochum, Germany; 10 Institute of Biochemistry, FAU Erlangen-Nürnberg, 91054 Erlangen, Germany

## Abstract

Oxidative stress (OS), mitochondrial dysfunction, and dysregulation of alpha-synuclein (aSyn) homeostasis are key pathogenic factors in Parkinson’s disease. Nevertheless, the role of aSyn in mitochondrial physiology remains elusive. Thus, we addressed the impact of aSyn specifically on mitochondrial response to OS in neural cells. We characterize a distinct type of mitochondrial fragmentation, following H_2_O_2_ or 6-OHDA-induced OS, defined by spherically-shaped and hyperpolarized mitochondria, termed “mitospheres”. Mitosphere formation mechanistically depended on the fission factor Drp1, and was paralleled by reduced mitochondrial fusion. Furthermore, mitospheres were linked to a decrease in mitochondrial activity, and preceded Caspase3 activation. Even though fragmentation of dysfunctional mitochondria is considered to be a prerequisite for mitochondrial degradation, mitospheres were not degraded via Parkin-mediated mitophagy. Importantly, we provide compelling evidence that aSyn prevents mitosphere formation and reduces apoptosis under OS. In contrast, aSyn did not protect against Rotenone, which led to a different, previously described donut-shaped mitochondrial morphology. Our findings reveal a dichotomic role of aSyn in mitochondrial biology, which is linked to distinct types of stress-induced mitochondrial fragmentation. Specifically, aSyn may be part of a cellular defense mechanism preserving neural mitochondrial homeostasis in the presence of increased OS levels, while not protecting against stressors directly affecting mitochondrial function.

Alpha-synuclein (aSyn) is an abundant cytosolic protein predominantly expressed in presynaptic neuronal terminals^1^, and has a central role in Parkinson’s disease (PD), the most prevalent neurodegenerative movement disorder. Multiplications and mutations in the SNCA genetic locus, encoding for aSyn, have been reported in inherited forms of PD (reviewed in Petrucci *et al*.^2^). Moreover, modified and aggregated aSyn forms are the major components of intraneuronal protein inclusions called Lewy bodies, the pathological hallmark of both familial and sporadic PD^3^. Interestingly, it was reported that brain regions of PD patients are not only characterized by aSyn-related pathology, but also mitochondrial impairment and high levels of oxidative stress (OS)^4^,^5^. This suggests that aSyn may have a specific functional role in mitochondrial homeostasis, with important implications for pathogenesis and disease progression in PD.

Mitochondria are a major source of reactive oxygen species (ROS), including superoxide anion (O_2_^−^) and hydrogen peroxide (H_2_O_2_), and reversely are highly vulnerable to OS-related damage^6^. Thus, mitochondrial dysfunction and OS may be reciprocally affected during disease progression^7^. Mitochondrial fission and fusion are crucial for quality control processes in the mitochondrial physiology^8^,^9^, which may also be affected in PD. Importantly, genes linked to autosomal recessive PD actively contribute to mitochondrial quality control. For example, PTEN Induced Putative Kinase 1 (PINK1) and Parkin commonly interact in a mitophagy pathway initiated by loss of the mitochondrial membrane potential (MMP)^10^,^11^,^12^.

Although increased levels of aSyn, especially in mutant or aggregated forms, have been linked to mitochondrial dysfunction (reviewed in Ryan *et al*.^7^), the physiological relevance of aSyn to mitochondrial quality control has not yet been clarified. Notably, some studies even suggested a protective role of wild type aSyn in the presence of OS^13^,^14^, however, without mechanistically specifying the aSyn effect on mitochondria. Given that mitochondria and OS are strongly interconnected, we hypothesized that aSyn modulates mitochondrial homeostasis and investigated its effect on mitochondrial response to OS in human neural cells. The study focuses on morphological changes of mitochondria, alterations in the MMP and function, and key components of mitochondrial quality control, namely fission, fusion, and Parkin-related mitophagy. In order to induce OS, H_2_O_2_ was chosen, since this ROS is also endogenously produced in cells, mainly via mitochondria. An excess of H_2_O_2_ inactivates mitochondrial energy production-related components^15^ and triggers the conversion to additional ROS, such as hydroxyl radicals or hydroxyl anions^16^. Furthermore, the H_2_O_2_ effects were compared to those of 6-OHDA and Rotenone, two synthetic oxidative stressors and mitochondrial toxins widely studied in the context of PD^17^,^18^,^19^.

A distinct type of mitochondrial fragmentation was observed, following H_2_O_2_ or 6-OHDA-induced OS in neuroglioma cells and neuronal cultures, defined by spherically-shaped and hyperpolarized mitochondria, which we have termed “mitospheres”. Mitosphere formation was dependent on the activity of the fission factor Dynamin-1-like protein (Drp1) and associated with a strong alteration of the mitochondrial fusion proteins, Mitofusin-1 (MFN1) and Optic atrophy 1 (OPA1), but not linked to an induction of Parkin-related mitophagy. Instead, mitospheres preceded Caspase3 activation and apoptosis. We noted that 6-OHDA also induced mitosphere formation and Caspase3 activation, whereas Rotenone did not result in these effects. Remarkably, aSyn prevented the formation of mitospheres and Caspase3 activation induced by H_2_O_2_ and 6-OHDA, but did not reduce the cellular susceptibility to Rotenone treatment. Our results identify a protective role of aSyn in mitochondrial homeostasis, specifically under OS-conditions that trigger mitosphere formation and Caspase3-dependent apoptosis.

## Results

### H_2_O_2_ induces the formation of “mitospheres” – spherically shaped and hyperpolarized mitochondria

**Figure 1:**
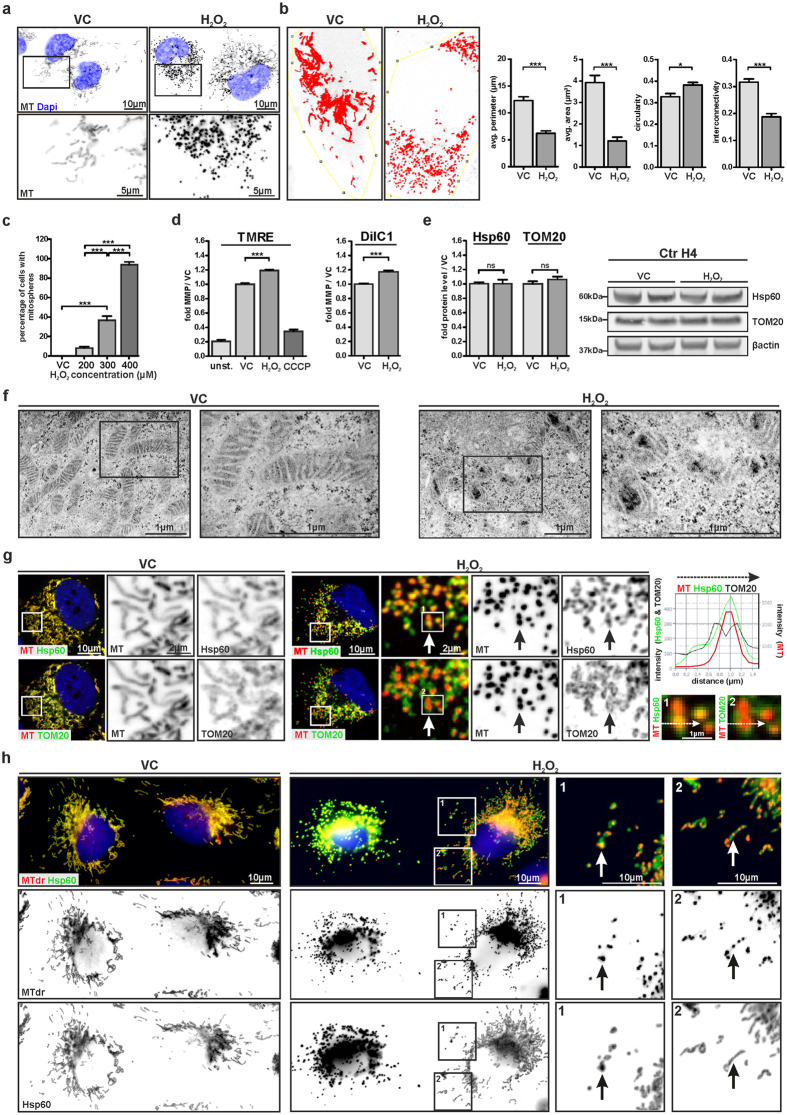
H_2_O_2_ induces the formation of mitospheres - spherically shaped and hyperpolarized mitochondria. (**a**) Human H4 neuroglioma cells were treated with 300 μM H_2_O_2_ or vehicle control (VC) for 4 h and the mitochondrial network was stained by MT. (**b**) The assessment of the average (avg.) perimeter, average area, circularity and interconnectivity of mitochondria from cells under VC-conditions and cells showing mitospheres under OS is shown (n = 9 cells analyzed per condition from three independent experiments, unpaired two-tailed t-test, avg. perimeter: p < 0.0001; avg. area: p < 0.0001; circularity: p = 0.0116; interconnectivity: p < 0.0001). (**c**) The percentage of cells with mitospheres increased with rising concentrations of H_2_O_2_, as quantified 4 h after treatment (One-way ANOVA Tukey’s Multiple Comparison Test, n = 3 with >300 cells quantified per sample, p < 0.0001, F = 242.2). (**d**) TMRE or DilC1 staining measured via flow cytometry showed that H_2_O_2_ exposure (300 μM, 4 h) led to an increase in MMP. Mean cellular fluorescence intensity compared to VC-treated cells is illustrated. CCCP-treatment served as control. unst. = unstained control (TMRE: One-way ANOVA Tukey’s Multiple Comparison Test, n ≥ 5, p < 0.0001 (VC vs. H_2_O_2_), DilC1: unpaired two-tailed t-test, n = 3, p = 0.0008). (**e**) H_2_O_2_ treatment did not influence Hsp60 and TOM20 levels as quantified via Western blot (WB) (unpaired two-tailed t-tests, n = 6, p = 0.9801 (Hsp60), p = 0.2853 (TOM20). (**f**) Electron microscopic analysis revealed that - in contrast to the tubular mitochondria under VC - H_2_O_2_-treated H4 cells show round-shaped mitochondria with electron-dense inclusions. (**g**,**h**) Mitochondria were stained with MT or MTdr, as well as antibodies against Hsp60 and TOM20. (**g**) Enlargements (1 and 2) representing the regions (marked by crisscross arrows) of the intensity profiles illustrate the co-labeling of mitospheres with Hsp60, as well as that the TOM20 staining surrounds the mitospheres. (**h**) Enlargement of different regions of the mitochondrial network within one cell shows parts of the mitochondrial network in which both MT and Hsp60 display spherical mitochondrial structures (1), while in other parts only the MMP-dependent MT accumulated in precise spherical regions (2).

In order to study the effects of OS on mitochondria, we exposed H4 neuroglioma cells to increasing concentrations of H_2_O_2_. H_2_O_2_ conferred substantial changes of the mitochondrial network (Fig. 1a). Particularly, we observed a specific change in the tubular mitochondrial morphology present under control conditions, characterized by the appearance of small, spherically-shaped mitochondria, which showed an increase in circularity and a reduction in the interconnectivity, the average perimeter, as well as in the average area (Fig. 1b). Moreover, the spherical morphology was paralleled by an intense MMP-dependent MitoTracker (MT) staining (Fig. 1a). These spherical mitochondria were clearly detectable 4 h after H_2_O_2_ treatment (Supplementary Fig. S1) and the percentage of cells showing this mitochondrial phenotype increased in a concentration dependent manner (Fig. 1c). In the following, we will refer to this specific mitochondrial phenotype as “mitospheres”. For 300 and 400 μM H_2_O_2_, ~40% and ~90% of cells, respectively, showed mitospheres after 4 h of treatment (Fig. 1c), yet, without a significant reduction in cell number (Supplementary Fig. S2a).

Both intra- and extracellular antioxidative mechanisms may lead to a reduction of the applied H_2_O_2_ concentration. Thus, to confirm that the treatment with H_2_O_2_ induced an increase in intracellular OS levels, we analyzed protein carbonylation, a marker for OS. We detected a significant increase in protein carbonylation (Supplementary Fig. S2b), reflecting a mild increase in OS levels due to H_2_O_2_ treatment. Importantly, the level of protein carbonylation was in a range comparable to brain lysates from aged mice (C57BL/6N, 50 weeks of age, Supplementary Fig. S2b).

The intense MT-staining prompted us to analyze whether mitosphere formation was accompanied by an altered MMP. Indeed, the mean cellular intensity of the MMP-sensitive dye TMRE was increased by ~20% after treating H4 cells with H_2_O_2_ (Fig. 1d). As control, cells were treated with the mitochondrial oxidative phosphorylation uncoupling agent carbonyl cyanide m-chlorophenyl hydrazone (CCCP), which almost abolished the TMRE signal. A similar H_2_O_2_-induced increase in MMP was also detected by using DilC1, another MMP-sensitive dye (Fig. 1d). At the same time, unaltered protein levels of Hsp60, a mitochondrial matrix protein, and TOM20, located in the outer mitochondrial membrane, indicated that H_2_O_2_-treatment did not result in changes of the total mass of mitochondria (Fig. 1e).

Further supporting these observations, ultrastructural analysis via electron microscopy revealed substantial changes of the mitochondrial network under OS. While vehicle control (VC)-treated cells showed tubular mitochondria with evenly distributed cristae, H_2_O_2_-treatment led to the formation of short and round-shaped mitochondria containing regions with electron-dense material (Fig. 1f).

Mitospheres were also positive for Hsp60 and TOM20 (Fig. 1g). Notably, spatial intensity distribution revealed a direct co-labeling of the mitochondrial matrix protein Hsp60 and the MT signal, while the staining of the outer mitochondrial membrane protein TOM20 surrounded the intensive MT- and Hsp60-positive spheres (Fig. 1g). Cells with pronounced H_2_O_2_-induced alterations displayed a complete replacement of the tubular mitochondrial network by mitospheres (Fig. 1h). Interestingly, in cells with a less altered mitochondrial network, the MT initially accumulated in precise spherical regions along the tubular mitochondrial network, whereas Hsp60 was still more evenly distributed and only partially accumulated in the mitospheres. This might be explained by the mitosphere-associated regional differences in the MMP, which influence the MT-staining.

### Mitosphere formation is a common cellular response to H_2_O_2_-induced oxidative stress

**Figure 2:**
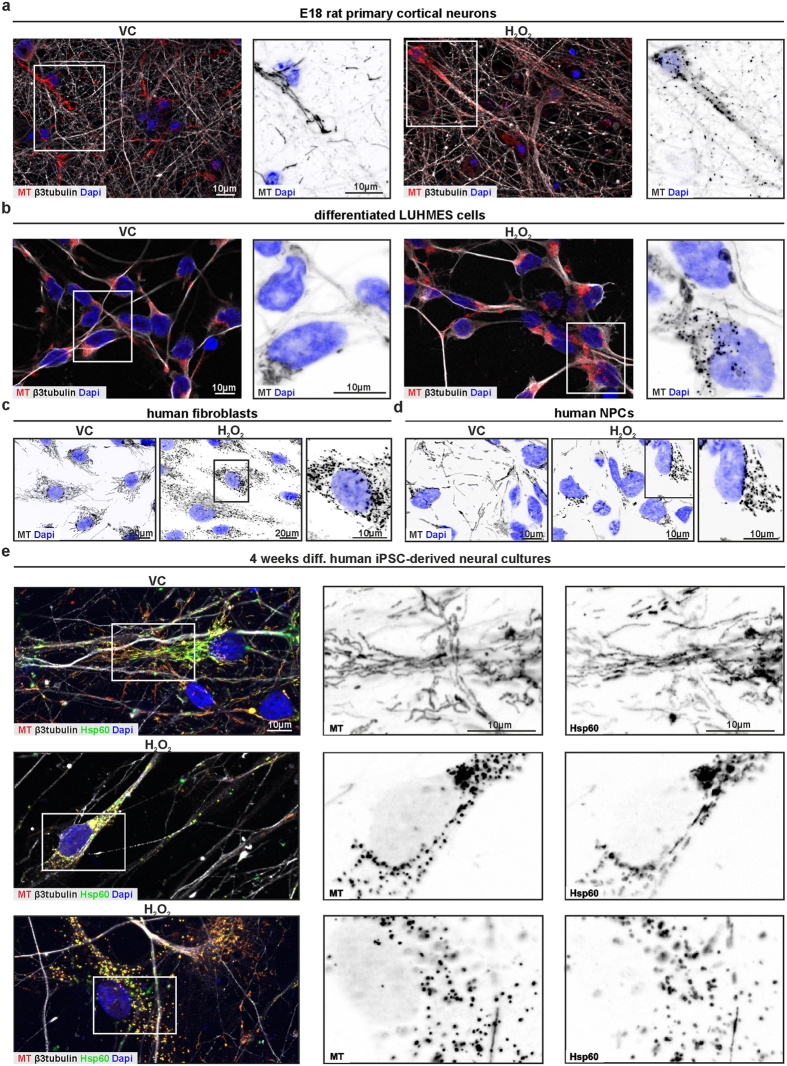
Mitosphere formation is a common cellular response to H_2_O_2_-induced oxidative stress. Differentiated E18 rat primary cortical neurons (DIV8) (**a**), 5 d differentiated LUHMES cells (**b**), human fibroblasts (**c**), human induced pluripotent stem cells (iPSC)-derived NPCs (**d**) and 4 weeks differentiated human iPSC-derived neural cultures (**e**) were treated with H_2_O_2_ for 4 h (primary neurons: 200 μM, LUHMES: 50 μM, fibroblasts: 100 μM, NPCs: 50 μM, 4 weeks diff. iPSC-derived neural cultures: 300 μM). Mitochondria were stained via MT. Neuronal processes of rat primary neurons, LUHMES cells and iPSC-derived neural cultures were visualized via β3-tubulin staining. Representative images of H_2_O_2_ concentrations leading to mitosphere formation without an apparent reduction in cell number are shown.

We next addressed whether the formation of mitospheres, described in H4 neuroglioma cells, is a common cellular response to H_2_O_2_-induced OS and also present in primary cells and neurons. Indeed, we detected mitospheres after H_2_O_2_-treatment in E18 rat primary cortical neurons differentiated for 8 d (DIV8) *in vitro* (Fig. 2a) and 5 d differentiated fetal human mesencephalic cells (LUHMES, Lund human mesencephalic) as human post-mitotic neuronal model (Fig. 2b). Consistently, human fibroblasts (Fig. 2c) and human neuronal precursor cells (NPCs) derived from induced pluripotent stem cells (iPSCs, Fig. 2d) also presented OS-related mitospheres. Moreover, in 4 weeks differentiated human iPSC-derived neural cultures (Fig. 2e), we observed H_2_O_2_-induced mitospheres in both, β3tubulin-positive neurons, as well as non-β3tubulin-positive cells with more glia-like morphology. These results confirm that mitosphere generation under OS was present in primary cells derived from rodent and human tissue, and in particular that mitospheres are also found in neurons. Notably, the H_2_O_2_ concentration required to induce mitospheres depended not only on cell type and cell density, but was also influenced by the composition of the culture medium (e.g. antioxidants) and ranged from 50 to 300 μM.

### H_2_O_2_-induced mitospheres form in a Drp1-dependent manner and are linked to changes in the levels of the fusion proteins MFN1 and OPA1

**Figure 3:**
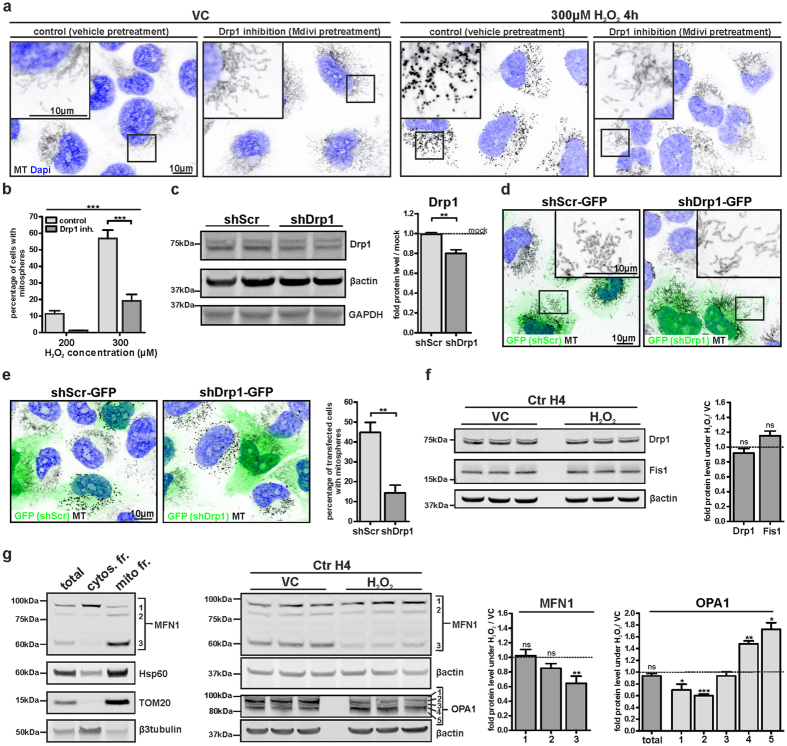
H_2_O_2_-induced mitospheres form in a Drp1-dependent manner and are linked to changes in the levels of the fusion proteins MFN1 and OPA1. (**a**) H4 cells were treated with H_2_O_2_ or VC for 4 h with (Drp1 inhibition) or without Mdivi pretreatment (control) and mitochondria were stained via MT. (**b**) The quantification of the percentage of cells with mitospheres shows that Drp1 inhibition substantially reduced mitosphere formation (Two-way ANOVA Bonferroni’s Multiple Comparison Test, n = 4 with >160 cells quantified per sample, F = 51.39, p < 0.0001 (control vs. Drp1 inhibition)). (**c**) H4 cells were transiently transfected with vectors expressing shDrp1 or a scrambled shRNA as control (shScr) and Drp1 levels were detected via WB. Considering a transfection efficiency of ~20–30%, the reduction in Drp1 protein levels in shDrp1 transfected cells by ~20% supports a strong downregulation of Drp1 in shRNA expressing cells. Fold protein levels per mock-transfected cells are shown (unpaired two-tailed t-test, n = 3, p = 0.0089). (**d**) H4 cells transfected with shDrp1-GFP constructs show more elongated mitochondria than control-transfected cells (shScr-GFP). Mitochondria were stained with MT. (**e**) Mitosphere formation was reduced in shDrp1-GFP expressing H4 cells treated with 300 μM H_2_O_2_ for 4 h as compared to shScr-GFP expressing control cells. (unpaired two-tailed t-test, n = 3 with >120 cells quantified per sample, p = 0.0086) (**f**) Protein levels of Drp1 and Fis1 were investigated via WB in H4 cells treated with VC or H_2_O_2_ (300 μM, 4 h; unpaired two-tailed t-tests, Drp1: n = 3, p = 0.3026; Fis1: n = 6, p = 0.1577). (**g**) Three MFN1 bands were observed in the mitochondrial fraction (Hsp60 ↑, TOM20 ↑, β3tubulin ↓) of H4 cells in a molecular weight range from 60 to 90 kDa. OS led to a strong reduction in the level of the MFN1 band detected at ~60 kDa (3) as analyzed in H4 cells exposed to 300 μM H_2_O_2_ for 4 h (unpaired two-tailed t-tests, n = 7, p = 0.8011 (1), p = 0.0866 (2), p = 0.004 (3)). H4 cells treated with 300 μM H_2_O_2_ for 4 h show a significant reduction in the levels of the long OPA1 isoforms (1 and 2) and an increase in the two short isoforms (4 and 5; unpaired two-tailed t-tests, OPA1: n = 3, p = 0.1749 (total), p = 0.0467 (1), p = 0.0001 (2), p = 0.5515 (3), p = 0.0081 (4), p = 0.0417 (5)).

Drp1, a member of the dynamin family of large GTPases, is a cytosolic protein that assembles onto mitochondria in order to facilitate organelle division and is thus a key molecule for mitochondrial fission^20^,^21^. To analyze whether the formation of mitospheres under OS depends on the mitochondrial fission machinery, we used both a pharmacological and a genetic approach. Mdivi is a chemical inhibitor of Drp1 that inhibits the self-assembly of Drp1 and thus blocks its activity^22^. Notably, the pretreatment of H4 cells with Mdivi strongly reduced H_2_O_2_-induced mitosphere formation as compared to vehicle-pretreated cells (Fig. 3a,b), supporting that mitosphere formation depends on the activity of the fission factor Drp1. Additionally, we used shRNA-mediated knockdown of Drp1 in order to specify the role of Drp1 for mitosphere formation. Drp1 levels in transiently transfected cells were reduced by ~20%, which supports a strong downregulation of Drp1 in cells expressing the shRNA, considering a transfection efficiency of ~20–30% (Fig. 3c). Moreover, the effectiveness of the shRNA against Drp1 was reflected by an elongated mitochondrial morphology of cells transfected with plasmids to express shDrp1 in combination with GFP, as compared to transfected cells expressing a scrambled shRNA control (Fig. 3d). In line with the results for Mdivi, the shRNA-mediated reduction in Drp1 levels significantly reduced the formation of mitospheres under H_2_O_2_ treatment (Fig. 3e), confirming that mitosphere formation depends on Drp1. However, the total levels of Drp1 and also Fis1, a Drp1-interacting factor localized at the outer mitochondrial membrane^23^,^24^, were not significantly altered under OS (Fig. 3f). Therefore, it is likely that mitosphere formation is triggered by an increase in the activity of the fission machinery, rather than an increase in the actual levels of the related fission proteins.

Additionally, we investigated the influence of OS on the levels of Mitofusin 1 (MFN1) and Optic atrophy 1 (OPA1), proteins that regulate the fusion of the mitochondrial outer and inner membrane, respectively^25^,^26^. We observed 3 bands for MFN1 in the mitochondrial fraction (Fig. 3g). Two of them were detected at ~85 kDa, and one at ~60 kDa. Interestingly, we found that H_2_O_2_ only led to a strong reduction in the level of the 60 kDa MFN1 isoform, which was most prominent in the mitochondrial fraction (Fig. 3g). Since OPA1 is expressed in eight mRNA splice forms, which also undergo post-translational proteolytic processing, a pattern of at least five bands (1–5) reflecting different OPA1 isoforms is usually detected via WB^27^. While the total OPA1 levels did not change, we found a significant reduction of two long OPA1 isoforms (bands 1 & 2, Fig. 3g), in combination with an increase in the levels of the two short OPA1 isoforms (bands 4 & 5) under OS. A reduction in the long OPA1 forms, in addition to the accumulation of short forms has been linked to fragmentation of mitochondria^28^,^29^. Changes in the levels of OPA1 and MFN1 may foster mitosphere formation by reducing refusion of these fragmented mitochondria.

### aSyn prevents H_2_O_2_-induced mitosphere formation

**Figure 4:**
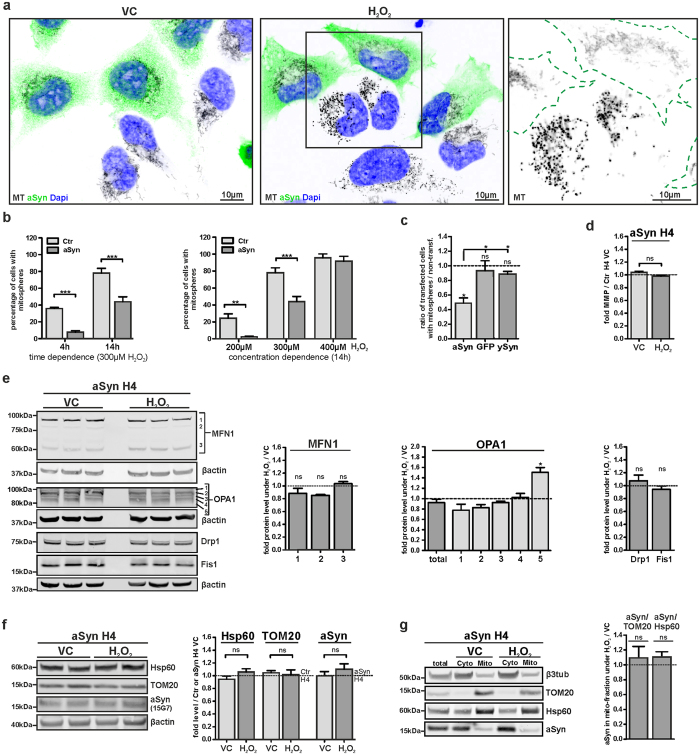
aSyn prevents H_2_O_2_-induced mitosphere formation. (**a**) Mixed cultured aSyn and Ctr H4 cells were treated with 300 μM H_2_O_2_ for 4 h. Immunofluorescence stainings for aSyn (15G7) and mitochondria (MT) are shown. (**b**) Quantification of the percentage of aSyn and Ctr H4 cells with mitospheres in a time- and concentration-dependent manner shows that aSyn overexpression substantially prevents mitosphere formation (Two-way ANOVA Bonferroni’s Multiple Comparison Test, n = 3 with >200 cells counted per sample, Ctr vs. aSyn: F = 51.69, p < 0.0001 (time dependence), F = 24.65, p = 0.0001 (concentration dependence)). (**c**) H4 cells transiently transfected with aSyn, GFP or γSyn were treated with 300 μM H_2_O_2_ for 4 h. Mitosphere formation was analyzed in transfected (20–30%) vs. non-transfected cells within the same cultures. Only aSyn-transfected cells showed a strong reduction in mitosphere formation (one-way ANOVA Dunnett’s Multiple Comparison Test, n = 3 with >600 cells counted per sample, p = 0.0266, F = 7.051, p = 0.0252 (aSyn vs. GFP), p = 0.0388 (aSyn vs. γSyn), DF = 6; one sample t-tests (two-tailed, theoretical mean = 1), p = 0.0193 (aSyn), p = 0.6621 (GFP), p = 0.0908 (γSyn)). (**d–g**) Stably aSyn overexpressing H4 cells were treated with 300 μM H_2_O_2_ for 4 h. (**d**) DilC1 staining measured via flow cytometry illustrates no differences in the MMP of aSyn H4 cells due to H_2_O_2_ exposure. Mean cellular fluorescence intensity compared to VC-treated Ctr H4 cells is illustrated (unpaired two-tailed t-test, n = 3, p = 0.0746). (**e)** H_2_O_2_ treatment had no influence on MFN1 levels (unpaired two-tailed t-tests, n = 3, p = 0.3627 (1), p = 0.0945 (2), p = 0.3913 (3)) or Drp1 and Fis1 levels (unpaired two-tailed t-tests, n = 6, Drp1: p = 0.4486; Fis1: p = 0.4315) in aSyn H4 cells. H_2_O_2_ treatment only led to an increase in the shortest OPA1 isoform (unpaired two-tailed t-tests, n = 3, p = 0.2958 (total), p = 0.1346 (1), p = 0.0744 (2), p = 0.3936 (3), p = 0.7992 (4), p = 0.0314 (5)) and (**f**) did not influence Hsp60, TOM20 and aSyn (15G7) levels (unpaired two-tailed t-tests, n = 6, p = 0.1431 (Hsp60), p = 0.7311 (TOM20), p = 0.3653 (aSyn)) in aSyn H4 cells as quantified via WB. (**g**) Isolations of crude mitochondrial fractions indicated a marginal increase in aSyn levels in the mitochondrial fractions due to H_2_O_2_ treatment (300 μM, 4 h), without reaching statistical significance. aSyn levels were normalized to TOM20 or Hsp60 levels (one-sample t-tests, theoretical mean = 1.0, n = 3, aSyn/TOM20: p = 0.6086; aSyn/Hsp60: p = 0.2716).

To clarify whether aSyn impacts the mitochondrial response to OS, we compared H_2_O_2_-induced mitosphere formation in stably aSyn-overexpressing (aSyn H4) and control H4 (Ctr H4) neuroglioma cells, which only express endogenous aSyn at a very low level. The usage of 1:1 mixed cell populations enabled us to simultaneously analyze aSyn H4 and control cells, thereby enabling direct comparability. Remarkably, aSyn overexpression strongly prevented mitosphere formation (Fig. 4a,b).

In order to confirm the observed differences in mitosphere formation detected in stably transduced H4 cells and to verify the specificity for aSyn, we used transient transfections of H4 cells with aSyn, GFP, and gamma-synuclein (γSyn) and quantified the percentage of cells with mitospheres in transfected (~20–30%) vs. non-transfected cells. All constructs were expressed under the CMV promoter with comparable transfection efficiency. In line with the observations in the stable cell lines, transient overexpression of aSyn prevented H_2_O_2_-induced mitosphere formation by ~50% (Fig. 4c). In contrast to aSyn, GFP and γSyn-overexpression did not influence mitosphere formation (Fig. 4c).

As for the Ctr H4 cells (compare Figs 1d and 3g), we analyzed the effects of H_2_O_2_ on MMP via flow cytometry, as well as on fusion by measuring OPA1 and MFN1 levels in aSyn H4 cells. To this end, we used a condition for which ~40% of Ctr H4, but only very few aSyn H4 cells displayed mitospheres (4 h treatment with 300 μM H_2_O_2_, Fig. 4b). In contrast to the Ctr H4 cells, MMP was not altered in aSyn H4 cells (Fig. 4d). Additionally, OS neither affected the level of the 60 kDa MFN1 isoform, nor the levels of the other two higher molecular weight MFN1 bands (~85 kDa) in aSyn H4 cells (Fig. 4e, for a direct comparison of OS-related changes in Ctr and aSyn H4 cells see WB in Supplementary Fig. S3). Similar to the Ctr H4 cells (compare Fig. 3g), OS reduced long OPA1 isoforms in aSyn H4 cells, while the levels of shorter OPA1 isoforms were increased. However, the effect was much less pronounced in aSyn H4, than in Ctr H4 cells and was only significant for the upregulation of the shortest of the detected isoforms (band 5, Fig. 4e). These findings further support the correlation of mitosphere generation to both a reduction in fusion and hyperpolarization of mitochondria. As for the Ctr H4 cells, we did not detect an impact of OS on the levels of the fission factors Fis1 and Drp1 (Fig. 4e). H_2_O_2_ also did not induce changes in the levels of Hsp60 and TOM20 in aSyn H4 cells (Fig. 4f), suggesting that mitochondrial mass was not altered. Moreover, no significant H_2_O_2_-induced changes in the aSyn levels were detected (Fig. 4f). The isolation of a crude mitochondrial fraction revealed a slight increase in the aSyn levels in the mitochondrial fractions under H_2_O_2_ treatment, when aSyn levels were normalized to TOM20 or Hsp60. Yet, this increase did not reach significance (Fig. 4g). Size exclusion chromatography was used to analyze the potential impact of H_2_O_2_ on aSyn oligomerization in aSyn H4 cells. For both VC and OS-condition the resulting retention times of aSyn species support the presence of monomeric aSyn^30^,^31^ and did not display a signal shift towards lower retention times corresponding to aSyn oligomers (Supplementary Fig. S2c). Moreover, we addressed the effect of H_2_O_2_ on aSyn aggregation by investigating the distribution of aSyn in different fractions (TBS-, TritonX-, RIPA-soluble vs. insoluble fractions) characterizing the solubility of aSyn. In line with the SEC, the solubility assay did not give evidence for an increase in aSyn aggregation (Supplementary Fig. S2d). Thus, under the conditions used, we did not find an OS-induced increase in aSyn oligomerization or aggregation.

### Increased aSyn expression reduces the formation of oxidative stress-induced mitospheres in neurons

**Figure 5:**
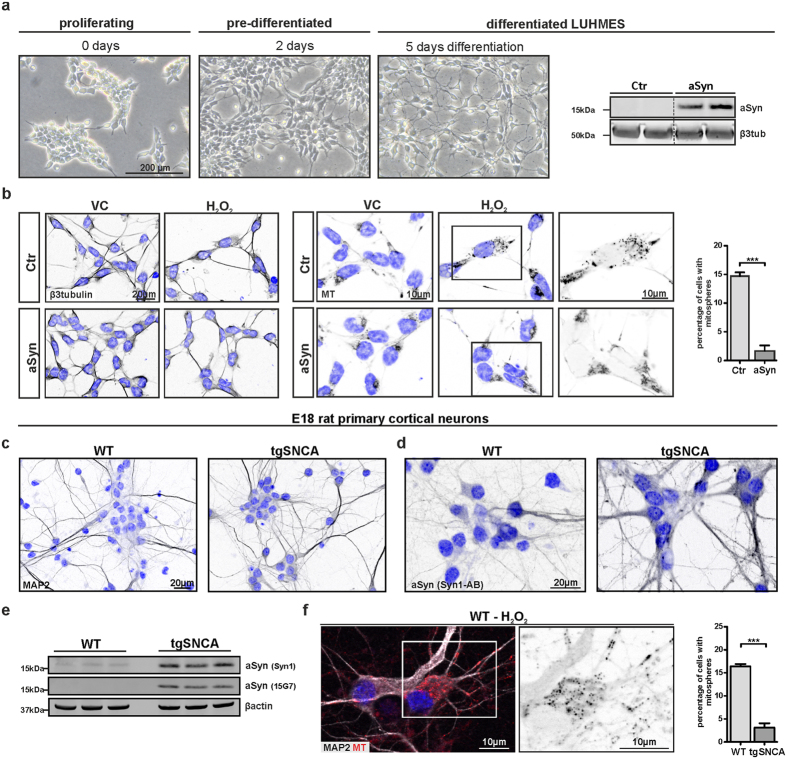
Increased aSyn expression reduces the formation of oxidative stress-induced mitospheres in neurons. (**a**) Proliferating, pre-differentiated and differentiated LUHMES cells visualized via light microscopy are depicted. aSyn (15G7) levels were detected by WB in differentiated Ctr and aSyn LUHMES cells. Bands shown for Ctr and aSyn LUHMES samples are derived from the same WB. (**b**) Differentiated LUHMES cells were treated with 50 μM H_2_O_2_ for 4 h and stained for β3-tubulin and with MT. Quantification of the percentage of cells with mitospheres revealed that aSyn overexpression prevented mitosphere formation in LUHMES cells (unpaired two-tailed t-test, n = 3 with >160 cells quantified per sample, p = 0.0004). (**c–f**) E18 rat primary cortical neurons of wild type (WT) or transgenic animals overexpressing human aSyn (tgSNCA) were cultured for 8 days *in vitro* (DIV8). (**c**) Immunocytochemical stainings for the neuronal marker MAP2 and (**d**) for aSyn (Syn1 antibody, detects human and rodent aSyn) are depicted. (**e**) WBs of aSyn detected via an antibody for both rodent and human aSyn (Syn1), as well as a human specific antibody (15G7) is shown. (**f**) Overexpression of human aSyn reduced mitosphere formation in cultures of primary cortical neurons, as quantified for cells treated with 200 μM H_2_O_2_ for 4 h (unpaired two-tailed t-test, n = 3 with >150 cells quantified per sample, p = 0.0002).

In order to validate our finding that aSyn reduces OS-related mitosphere formation in human neurons, we used differentiated stably aSyn-overexpressing (aSyn LUHMES) and control LUHMES (Ctr LUHMES) cells (Fig. 5a) that display a very homogeneous neuronal phenotype and analyzed the mitochondrial response to OS. As compared to H4 cells, differentiated LUHMES cells showed in general a higher susceptibility to H_2_O_2_. Indeed, mitosphere formation was already detectable after the treatment with 50 μM H_2_O_2_ for 4 h. In line with the results that we obtained by using H4 cells, aSyn overexpression significantly reduced the percentage of cells with mitospheres in LUHMES cells (Fig. 5b). In order to address, the influence of human aSyn on mitochondria under OS in primary neuronal cultures, we studied the effect of H_2_O_2_ on mitosphere formation in rat primary cortical neurons derived from wild type animals (endogenous aSyn levels) in comparison to primary neurons from transgenic animals overexpressing human aSyn (Fig. 5c–e). Intriguingly, when treating primary neurons with 200 μM H_2_O_2_, a condition for which mitospheres were frequently found in neurons from wild type animals (~15%), we detected a strong reduction in mitosphere formation in cells from transgenic animals (Fig. 5f).

These results support that aSyn overexpression also reduces mitosphere formation in neurons and that increased aSyn levels induce a protective effect on mitochondrial homeostasis under OS.

### aSyn potentiates mitochondrial fragmentation under basal conditions

**Figure 6:**
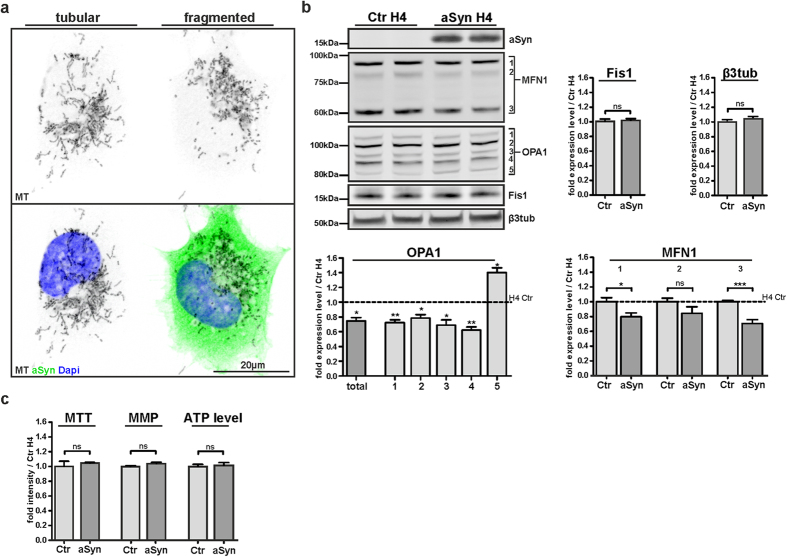
aSyn potentiates mitochondrial fragmentation under basal conditions. (**a**) Immunocytochemical staining of aSyn (15G7) and mitochondria (MT) of mixed cultured aSyn and Ctr H4 cells shows more fragmented mitochondria in aSyn overexpressing cells. (**b**) Quantification of the protein levels of OPA1, MFN1 and Fis1, as well as β3-tubulin levels as controls, indicates an influence of aSyn overexpression on mitochondrial fusion proteins (unpaired two-tailed t-tests, OPA1: n = 5, p = 0.0114 (total), p = 0.0027 (1), p = 0.0158 (2), p = 0.0245 (3), p = 0.0015 (4), p = 0.0136 (5), MFN1: n = 10, p = 0.0155 (1), p = 0.1348 (2), p < 0.0001 (3), Fis1: n = 5, p = 0.7619, β3-tubulin: n = 10, p = 0.3295). (**c**) Measurement of cell viability via MTT assay (unpaired two-tailed t-test, n = 3, p = 0.5440), of MMP via DilC1 staining and flow cytometry (unpaired two-tailed t-test, n = 3, p = 0.1313), as well as of ATP levels (unpaired two-tailed t-test, n ≥ 4, p = 0.7604) showed no differences between aSyn and Ctr H4 cells.

OS-induced mitosphere formation reflects a strong fragmentation of the mitochondrial network and we observed that aSyn substantially reduced the formation of mitospheres. Interestingly, previous studies reported that overexpression of aSyn itself increases mitochondrial fragmentation^32^,^33^. Notably, under basal conditions, we also observed that aSyn H4 cells showed a more fragmented morphology of mitochondria (Fig. 6a). However, the aSyn-dependent fragmentation revealed short mitochondria that differed morphologically from the round-shaped OS-induced mitospheres intensively stained by MT. In order to address the related mechanisms of this aSyn-dependent effect on mitochondrial fragmentation, we analyzed the influence of aSyn on key proteins relevant for the fission and fusion of mitochondria. Although aSyn prevented the strong OS-induced reduction of the 60 kDa MFN1 band (Figs 3g and 4e and Supplementary Fig. S3), we observed that aSyn overexpression itself led to a reduction in MFN1 levels, including both one of the two higher molecular weight MFN1 bands (~85 kDa) and also the 60 kDa band under basal conditions (Fig. 6b), suggesting a general reduction in fusion of mitochondria. Moreover, the total level of OPA1 (Fig. 6b) was significantly reduced in aSyn H4 cells. A separate evaluation of the five different OPA1 bands (Fig. 6b) illustrated that aSyn overexpression led to a decrease in the expression of band 1–4, but increased the level of band 5. Since a reduction in the long OPA1 forms, in addition to the accumulation of short forms has been linked to fragmentation of mitochondria^28^,^29^, the aSyn-based changes in the levels of the different OPA1 isoforms are in line with a reduction in fusion of mitochondria. In contrast to MFN1 and OPA1, we did not find an alteration in the level of the fission factor Fis1 due to aSyn overexpression (Fig. 6b). Notably, the changes in mitochondrial fusion due to aSyn overexpression were not associated with alterations in cellular viability, MMP and ATP levels (Fig. 6c).

### Mitosphere formation is not linked to Parkin-related mitophagy

**Figure 7:**
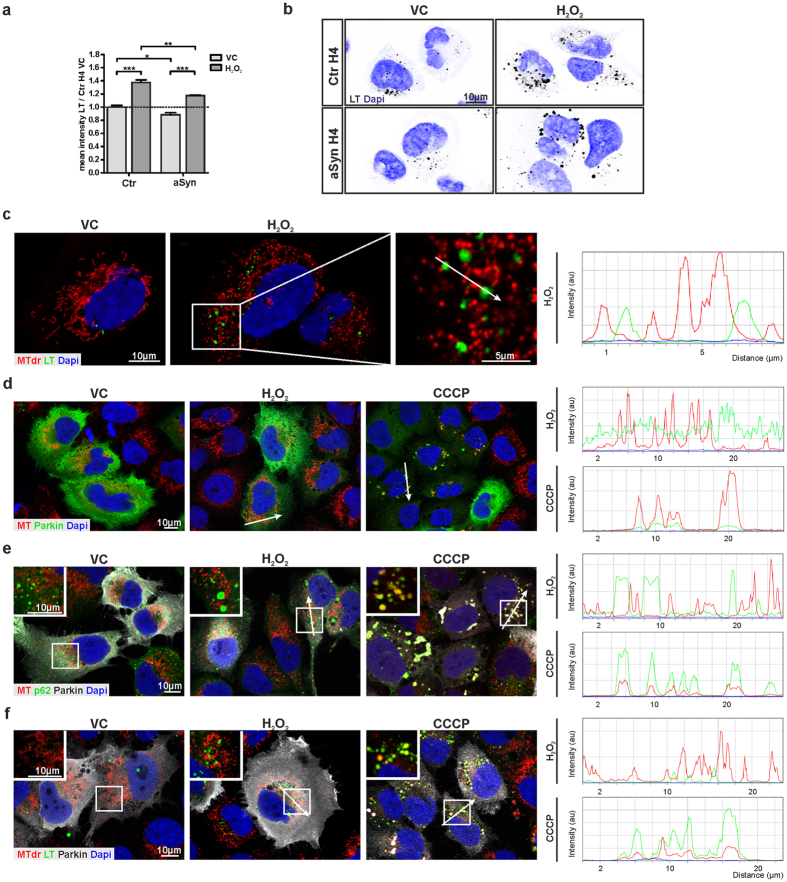
Mitosphere formation is not linked to Parkin-related mitophagy. (**a**,**b**) OS led to an upregulation of LysoTracker Red (LT)-positive structures as investigated via flow cytometry (**a**) and fluorescence microscopy (**b**) in aSyn and Ctr H4 cells treated with 300 μM H_2_O_2_ for 4 h. (**a**) The relative mean cellular intensity of the LT signal compared to VC-treated Ctr H4 cells is shown (Two-way ANOVA Bonferroni’s Multiple Comparison Test, n = 3, F = 131.5, p < 0.0001 (VC vs. H_2_O_2_); F = 29.68, p = 0.0006 (Ctr vs. aSyn)). (**c**) MTdr-stained mitospheres did not show co-localization with LT-positive organelles as investigated via fluorescence microscopy 4 h after the treatment with 300 μM H_2_O_2_. Intensity profiles, along the white arrows, of the MTdr (red) and LT (green) staining are depicted. (**d–f**) H4 cells transiently overexpressing Parkin were treated for 4 h with 300 μM H_2_O_2_ or 50 μM CCCP as control. Parkin (**d**) and p62 recruitment (**e**) as well as co-localization of mitochondria and LT-positive organelles (**f**) was investigated via fluorescence staining. In contrast to mitochondria of CCCP-treated cells, H_2_O_2_-induced mitospheres did not show co-labeling with Parkin, p62 or LT. The red color of the intensity profiles along the white arrows reflects the MT or MTdr staining, the green color represents Parkin, p62 and LT staining in (**d**–**f**) respectively.

In order to analyze the mechanistic basis of mitosphere formation, we addressed whether they are part of a degradative pathway involved in mitochondrial quality control. The lysosomal compartment has been reported to play an important role in the detoxifying defense against OS via the removal of dysfunctional mitochondria^34^,^35^,^36^. Hence, we investigated whether the OS-related mitospheres co-localize with the lysosomal compartment. Interestingly, although the LysoTracker Red (LT)-positive compartment was upregulated in both Ctr and aSyn H4 cells after H_2_O_2_-treatment (Fig. 7a,b), mitospheres did not show a substantial co-localization with the LT-staining (Fig. 7c).

We found that mitospheres form in a Drp1-dependent manner and are paralleled by a strong reduction in the 60 kDa MFN1 band. Interestingly, both Drp1-activity and also the proteasomal degradation of MFN1 are prerequisites for ubiquitin ligase Parkin-mediated mitophagy^37^. Thus, we further analyzed whether mitospheres are linked to Parkin-dependent mitophagy, by analyzing whether overexpression of Parkin stimulates the degradation of mitospheres via autophagy-related pathways. Overexpression of Parkin has been shown to accelerate mitophagy of dysfunctional mitochondria in the presence of CCCP, a compound that directly affects the membrane potential of mitochondria^38^. Immunofluorescence staining of Parkin-transfected H4 cells demonstrated that Parkin is not recruited to OS-related mitospheres (Fig. 7d, Supplementary Fig. S4a,b). In contrast, under CCCP-treatment Parkin recruitment to mitochondria was observed after 30 min and even stronger after 4 h (Fig. 7d, Supplementary Fig. S5a,b). Moreover, under CCCP-treatment Parkin-overexpression led to a strong co-localization of mitochondria and p62, a protein involved in the recruitment of the autophagy machinery during mitophagy^39^,^40^. However, p62 did not substantially co-localize with mitospheres under H_2_O_2_-treatment (Fig. 7e, Supplementary Figs S4a and 5a). Similarly, Parkin did not stimulate the co-localization of mitochondria with LT-positive compartments under H_2_O_2_-treatment (Fig. 7f). Notably, after 8 h of treatment with CCCP, recruitment of Parkin and p62 to mitochondria was not detectable anymore (Supplementary Fig. S5a,b). Yet, MT staining (from 8 h onwards) and Hsp60 staining (from 24 h onwards) was lost in Parkin-transfected cells (Supplementary Fig. S5b), supporting that Parkin overexpression triggers the degradation of mitochondria under CCCP treatment. In contrast, under H_2_O_2_, mitochondria were not cleared and mitospheres were still present in Parkin-transfected cells, even after 40 h (Supplementary Fig. S4b). In total, these findings indicate that mitospheres are not degraded by lysosomal-associated pathways, including Parkin-mediated mitophagy.

### aSyn reduces Caspase3 activation and protects against oxidative stress

**Figure 8:**
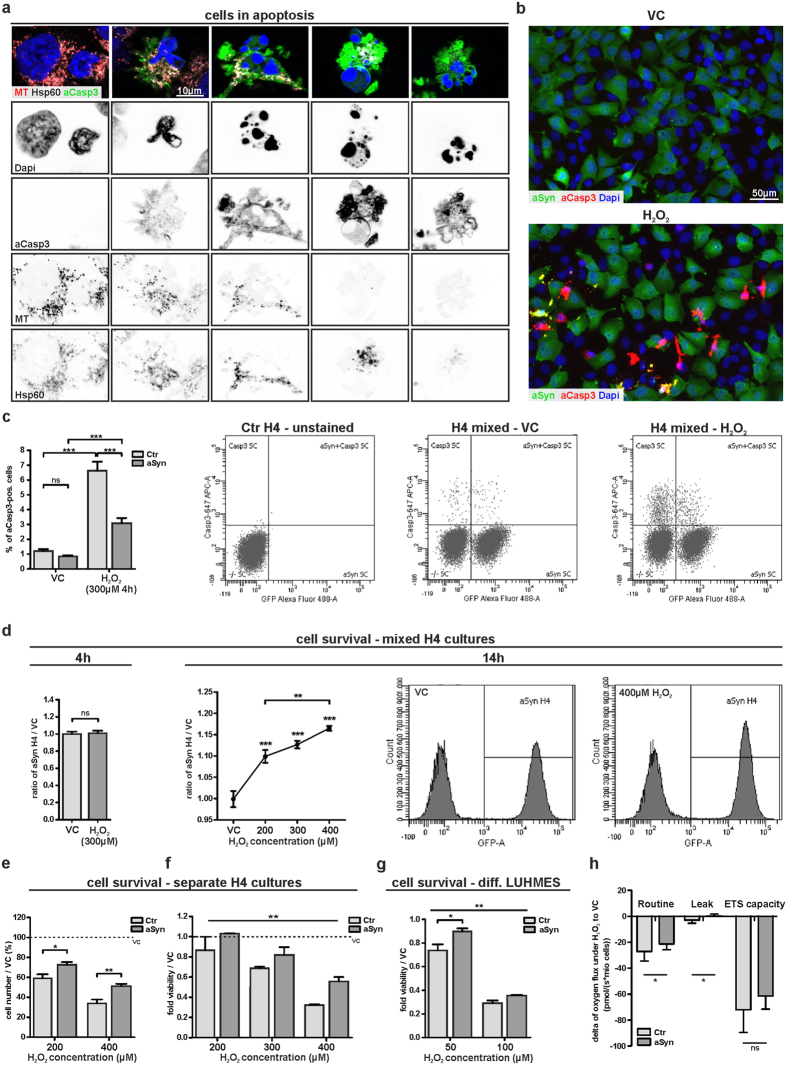
aSyn reduces Caspase3 activation and protects against oxidative stress. (**a–c**) H4 cells were treated with 300 μM H_2_O_2_ for 4 h. (**a**) aCasp3 as well as mitochondria (Hsp60 and MT) were visualized in different stages of apoptosis. (**b**,**c**) The assessment of Casp3 activation in a mixed culture of aSyn and Ctr H4 cells via immunocytochemistry (**b**) and flow cytometry (**c**) revealed that aSyn overexpression led to a reduction of H_2_O_2_-related Casp3 activation. (**c**) Two-way ANOVA (randomized block experiments) Bonferroni’s Multiple Comparison Test, n = 11, Ctr vs. aSyn: VC t = 1.471, H_2_O_2_ t = 14.68, DF = 20; Scatter blots representing GFP (aSyn H4) and aCasp3 (647) fluorescence signals of single cells (SC) as measured via flow cytometry are depicted. (**d**) The ratio of aSyn H4 cells after H_2_O_2_-treatment in comparison to VC-treated cells was analyzed in mixed cultured aSyn and Ctr H4 cells via flow cytometry. While there was no difference in the relative cell number of both cell populations 4 h after treatment with 300 μM H_2_O_2_, the ratio of aSyn H4 cells increased after 14 h with rising H_2_O_2_ concentrations (competitive survival assay (CSA), 4 h: unpaired two-tailed t-test, n = 11, p = 0.8514; 14 h: One-way ANOVA Tukey’s Multiple Comparison Test, n ≥ 6, F = 27.68, p < 0.0001). (**e**,**f**) The measurement of cell viability in separate cultures of aSyn and Ctr H4 cells 14 h after H_2_O_2_-treatment via cell counting (**e**, Two-way ANOVA Bonferroni’s Multiple Comparison Test, n = 4, F = 13.99, p = 0.0015 (Ctr vs. aSyn)) and MTT assay (**f**, Two-way ANOVA Bonferroni’s Multiple Comparison Test, n = 3, F = 9.221, p = 0.0079 (Ctr vs. aSyn)) revealed that aSyn H4 cells are more resistant to OS. (**g**) Ctr and aSyn LUHMES cells were treated with 50 or 100 μM H_2_O_2_ for 4 h. Analysis of cell viability via MTS assay showed that aSyn overexpression reduced the susceptibility to OS in LUHMES cells (Two-way ANOVA Bonferroni’s Multiple Comparison Test, n = 3, F = 12.87, p = 0.0071 (Ctr vs. aSyn)). (**h**) Oxygen consumption was measured in VC- or H_2_O_2_ (300 μM, 4 h)-treated Ctr and aSyn H4 cells. Delta of oxygen flux for H_2_O_2_-treated cells compared to control conditions is shown for Routine respiration, Leak respiration, and maximum respiration (electron transport system (ETS) capacity; unpaired two-tailed t-tests, n = 5, Routine: p = 0.0464; Leak: p = 0.0105, ETS: p = 0.1119).

Since OS may trigger apoptosis^41^, we asked whether the formation of mitospheres is linked to the activation of apoptotic pathways. Thus, we investigated the activation of Caspase3. Although we did not detect significant cell loss 4 h after the treatment with 300 μM H_2_O_2_ (Supplementary Fig. S2a), we found an increased number of activated Caspase3 (aCasp3)-positive cells (Fig. 8a,b). Furthermore, we observed that the aCasp3-positive cells that still showed a positive MT signal displayed mitospheres, suggesting that mitosphere formation precedes Caspase3 activation (Fig. 8a). Interestingly, during the progression of apoptosis the MMP-dependent MT signal disappeared earlier than the staining of the mitochondrial matrix protein Hsp60. This suggests that the hyperpolarized state of mitochondria is followed by a loss of the MMP before mitochondrial structures are completely degraded.

To investigate the effect of aSyn overexpression on Casp3-activation under OS, we compared the percentage of aCasp3-positive aSyn H4 to Ctr H4 cells in mixed cultures. Notably, aSyn overexpression reduced the percentage of H_2_O_2_-induced aCasp3-positive cells (Fig. 8b,c). Moreover, we analyzed whether aSyn overexpression also affected the cellular resistance to OS. Therefore, we performed a competitive survival assay (CSA) that enabled us to analyze the relative cellular survival under OS: 1:1 mixed cultures of aSyn and Ctr H4 cells were treated for 4 h or 14 h with H_2_O_2_ and the percentage of aSyn H4 cells in comparison to VC-treated cells was determined. While we did not detect differences in the relative survival of both cell populations 4 h after treatment with 300 μM H_2_O_2_, we found that after 14 h the ratio of aSyn H4 cells increased with rising H_2_O_2_ concentrations, revealing that aSyn promotes cellular resistance to H_2_O_2_-induced OS (Fig. 8d). In accordance to the findings in the mixed cultures, we found a reduced susceptibility of aSyn H4 cells to H_2_O_2_ in separate cultures of aSyn and Ctr H4 cells, by quantifying the absolute cell number, as well as by using MTT assay (Fig. 8e,f).

Furthermore, we analyzed the susceptibility to OS in differentiated neurons by using LUHMES cells and found that cell viability was significantly less affected in aSyn LUHMES cells after H_2_O_2_-treatment, as compared to controls (Fig. 8g), suggesting that aSyn also reduces the susceptibility to OS in neurons. In total, these findings demonstrate that aSyn has the ability to prevent mitosphere formation, which is linked to Caspase3 activation, and therefore protects against OS.

Dysfunctional mitochondria may directly trigger apoptotic pathways and the changes in the MMP under H_2_O_2_ treatment suggest that mitosphere formation is accompanied by an altered mitochondrial function. Therefore, we performed mitochondrial respirometry measurements and analyzed the impact of the applied OS-conditions on oxygen consumption in both Ctr and aSyn H4 cells. We found a significant reduction in the oxygen flux due to H_2_O_2_ treatment (Fig. 8h), indicating a decrease in mitochondrial activity. While we did not detect differences in the oxygen flux under control conditions between Ctr and aSyn H4 cells (oxygen flux (pmol/(s*mio cells)), mean + SD (Ctr H4) = 48.88 ± 6.71, mean + SD (aSyn H4) = 49.77 ± 4.98, n = 5, p = 0.7383), overexpression of aSyn attenuated the reduction in oxygen consumption observed under H_2_O_2_ (Fig. 8h). This suggests that the protective effect of aSyn under OS is - at least partially - mediated via stabilizing mitochondrial function.

### aSyn prevents mitosphere formation and Caspase3 activation induced by 6-OHDA

**Figure 9:**
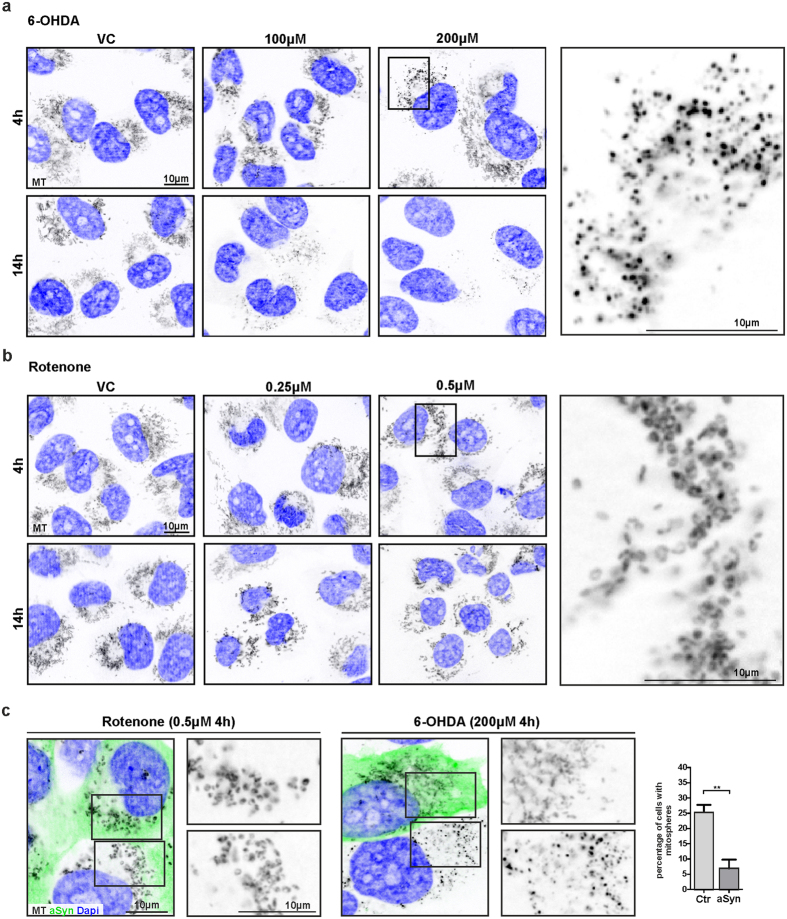
aSyn prevents mitosphere formation induced by 6-OHDA. (**a**,**b**) H4 cells were treated with different concentrations of 6-OHDA (**a**) or Rotenone (**b**) for 4 or 14 h and mitochondrial alterations are shown via MT staining. (**c**) Whereas the “donut-shaped” mitochondrial alterations induced by Rotenone were also frequently found in aSyn H4 cells, aSyn overexpression significantly reduced 6-OHDA-induced mitosphere formation. Quantification of mitospheres under 6-OHDA treatment (200 μM, 4 h) was performed in mixed cultures of aSyn and Ctr H4 cells (unpaired two-tailed t-test, n = 3 with >600 cells counted per sample, p = 0.0086).

In addition to H_2_O_2_, we used 6-Hydroxydopamine (6-OHDA) and Rotenone as direct or indirect inducers of OS^17^,^19^,^42^. 6-OHDA, which leads to a general increase in ROS levels, resulted in mitosphere formation 4 h after treatment, similar to H_2_O_2_ (Fig. 9a). In addition to the mitosphere formation, 6-OHDA also appeared to induce a loss of MMP which predominantly occurred after longer treatment periods (14 h, Fig. 9a). In contrast, Rotenone, which mainly fulfills its toxic function by inhibiting complex I of mitochondria^17^,^43^, did not induce mitosphere formation, but “donut-shaped” mitochondrial structures (Fig. 9b), which appeared not to be influenced by aSyn overexpression (Fig. 9c). Importantly, the 6-OHDA-induced mitosphere formation was strongly reduced by aSyn overexpression (Fig. 9c), similar to the observations under H_2_O_2_ treatment (Fig. 4b).

**Figure 10:**
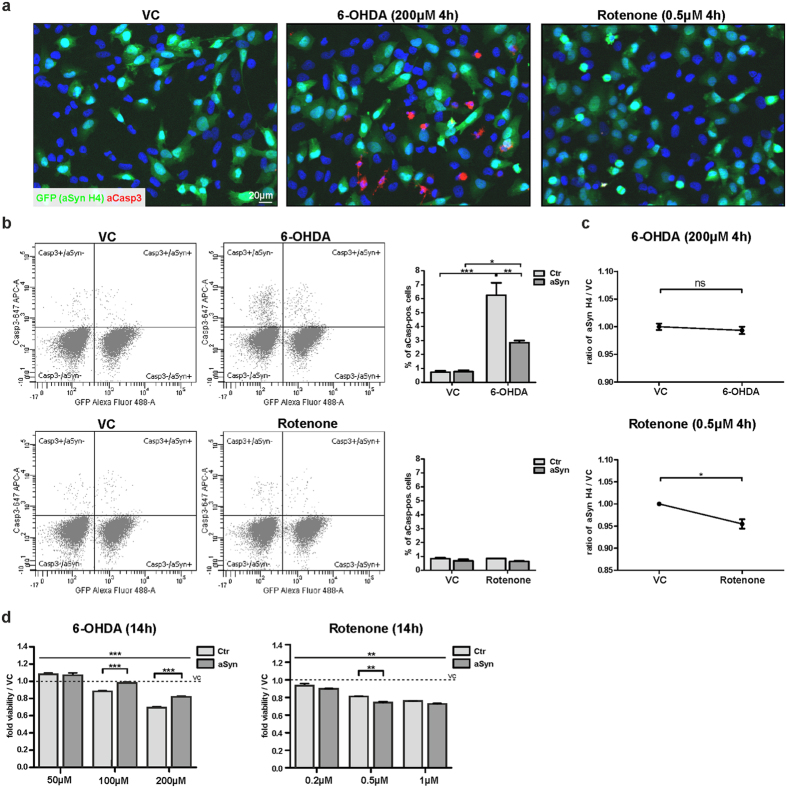
aSyn prevents Caspase3 activation induced by 6-OHDA. (**a**) Mixed cultures of Ctr and aSyn H4 cells were treated with 6-OHDA and Rotenone as indicated and stained for aCasp3. (**b**) Flow cytometry revealed an increase in Casp3 activation under 6-OHDA (200 μM, 4 h), but not under Rotenone (0.5 μM, 4 h) treatment. aSyn overexpression reduced the activation of Caspase3 under 6-OHDA (200 μM, 4 h). Scatter blots representing GFP (aSyn H4) and aCasp3 (647) fluorescence signals of single cells (SC) as measured via flow cytometry are depicted (Two-way ANOVA Bonferroni’s Multiple Comparison Test, 6-OHDA: n = 3, Ctr vs. aSyn: VC t = 0.0614 (p > 0.05), 6-OHDA-treatment t = 5.216 (p < 0.01), Rotenone: n = 3, Ctr vs. aSyn: VC t = 1.461 (p > 0.05), Rotenone-treatment t = 2.125 (p > 0.05)). (**c**) The ratio of aSyn H4 cells after exposure to 6-OHDA or Rotenone in comparison to VC-treated cells was analyzed in mixed cultured aSyn and Ctr H4 cells via flow cytometry. While there was no difference in the relative cell number of both cell populations 4 h after the treatment with 6-OHDA, Rotenone-treatment led to a reduction in the ratio of aSyn H4 cells (CSA, unpaired two-tailed t-tests, 6-OHDA: n = 3, p = 0.4918; Rotenone: n = 3, p = 0.0138). (**d**) The measurement of cell viability in separate cultures of aSyn and Ctr H4 cells 14 h after 6-OHDA and Rotenone treatment via MTS assay revealed that aSyn H4 cells are more resistant to 6-OHDA, but not to Rotenone (Two-way ANOVA Bonferroni’s Multiple Comparison Test, 6-OHDA: n = 3, F = 30.40, p < 0.0001 (Ctr vs. aSyn), Rotenone: n = 3, F = 15.66, p = 0.0011 (Ctr vs. aSyn)).

In line with this, increased Caspase3 activation was observed after 4 h of 6-OHDA treatment (Fig. 10a), which was also reduced by aSyn overexpression (Fig. 10b) and associated with an aSyn-dependent attenuation of the impaired cell viability after 14 h (Fig. 10c,d). Although, the reduction in cell viability in control cells was comparable for the concentrations used for 6-OHDA and Rotenone, Rotenone treatment did not induce Caspase3 activation (Fig. 10a,b). Notably, aSyn did not reduce the cellular susceptibility to Rotenone treatment (Fig. 10c,d). On the contrary, we even found a reduction of aSyn cells assessed by CSA after 4 h of Rotenone treatment and aSyn overexpression rather increased cellular toxicity measured after 14 h of Rotenone exposure.

Although we found that the beneficial effect of aSyn overexpression depended on the individual cell stressor, immunocytochemical stainings of aSyn under both 6-OHDA and Rotenone did not reveal hints for an induction of aSyn aggregation under the conditions used here (Supplementary Fig. S6).

## Discussion

Both OS and mitochondrial dysfunction represent key factors crucial for the progression of PD^5^. Although the relevance of aSyn for PD is well-recognized, it emerged to be quite challenging to clarify the contribution of aSyn to the interplay of OS and mitochondrial dysfunction. Here, we addressed the impact of aSyn on components essential for mitochondrial homeostasis, including mitochondrial morphology, fission and fusion proteins, mitochondrial function and apoptosis under OS.

**Figure 11:**
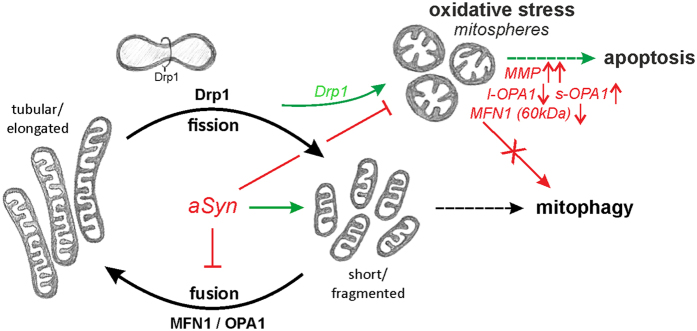
Summary of the impact of aSyn on mitochondrial dynamics. Both fission and fusion enable dynamic morphological changes of the mitochondrial network contributing to mitochondrial maintenance, function and quality control^97^. While MFN1 and OPA1 mediate the fusion of the mitochondrial outer and inner membrane, respectively, Drp1 is a key factor in mitochondrial fission. During mitochondrial fission, cytosolic Drp1 is recruited to mitochondrial outer membrane, wraps around tubular mitochondria leading to constriction and division of the organelles. Fission processes result in the generation of short/fragmented mitochondria and thus facilitate the degradation via autophagy-lysosomal pathways (mitophagy). We found that OS induced via H_2_O_2_ led to the formation of mitospheres, hyperpolarized mitochondria with a punctate shape that differ from short/fragmented mitochondria that are also present under basal conditions. Mitospheres form in a Drp1-dependent manner and are linked to changes in the levels of the fusion proteins MFN1 (60 kDa band) and OPA1 (l-OPA1: long OPA1 isoforms, s-OPA1: short OPA1 isoforms). Their formation is associated with a reduced mitochondrial function, but mitospheres are not degraded via Parkin-mediated mitophagy. Instead, they precede Caspase3 activation and apoptosis. An increase in shorter mitochondria due to aSyn expression may be attributable to the impact of aSyn on the levels of the fusion proteins MFN1 and OPA1. Intriguingly, while aSyn appears to promote mitochondrial fragmentation under basal conditions, our results reveal that it inhibits mitosphere formation and apoptosis under OS.

We report that OS results in a specific form of mitochondrial fragmentation. Particularly, distinct from classical fragmentation, mitochondria displayed a spherical and hyperpolarized phenotype, which we termed “mitospheres”. Mitosphere formation was dependent on the fission factor Drp1 and paralleled by profound changes in the levels of the fusion proteins MFN1 and OPA1. It was not related to Parkin-mediated mitophagy, but linked to a reduction in oxygen consumption and preceded Casp3 activation and apoptosis. Intriguingly, we found that aSyn modulates mitochondrial fusion and substantially prevents the generation of OS-related mitospheres, accompanied by a reduction in apoptosis (Fig. 11).

In order to cause an increase in OS levels, we applied H_2_O_2,_ a molecule that is also endogenously produced in cells^15^,^16^. Additionally, we compared the effects of H_2_O_2_, to those of 6-OHDA and Rotenone, which generally increase ROS levels and/or mediate direct effects on mitochondria via complex I inhibition^17^,^19^,^42^,^44^. Mitosphere formation and Caspase3 activation was detected for both H_2_O_2_ and 6-OHDA treatment, but not under Rotenone exposure that led to a donut-shaped mitochondrial morphology, as previously described^43^. Interestingly, this correlated with the beneficial effect of aSyn overexpression, which was only protective under H_2_O_2_ and 6-OHDA, but not in the presence of Rotenone.

The appearance of mitospheres, which was clearly detectable 4 h after the treatment with H_2_O_2_ or 6-OHDA, was identified in differentiated neurons and several other cell types. We observed mitosphere formation in a considerable proportion of H4 cells (30–45%) under OS-conditions (4 h, 300 μM H_2_O_2_), for which we did not detect substantial cell loss and only a mild increase in aCasp3-positive cells (4–9%). H_2_O_2_ led to an increase in protein carbonylation in H4 cells, comparable to aged mouse brain tissue, confirming a gentle raise in intracellular OS levels. Mitosphere formation (detected for H_2_O_2_ concentrations ranging from 50–300 μM) and the susceptibility of the different cells to the applied H_2_O_2_ concentrations may not only depend on the cell type and the cell density, but also on the composition of the culture medium. Although it is likely that only a minor part of the applied H_2_O_2_ contributed to the increase in intracellular OS levels, it is interesting to note that H_2_O_2_ concentrations in a comparable range were described in brain tissue under pathological conditions^45^.

Ultrastructural analysis revealed that mitospheres comprise regions with electron-dense material, but do not show a substantial reorganization of the cristae structure. A general increase in mitochondrial fragmentation under OS has been described in different cell types of rodent and human origin, including neurons^46^,^47^,^48^,^49^. Intriguingly, OS-induction by nitric oxide (NO) or 3-Nitropropionic acid (3-NP) also led to the formation of punctate mitochondria in rat primary cortical neurons, similar to the H_2_O_2_ or 6-OHDA-induced mitospheres observed in the present study^46^,^47^.

In contrast to H_2_O_2_ and 6-OHDA, Rotenone treatment did not lead to mitosphere formation. Here, the predominant mitochondrial alterations are reflected via “donut shaped” structures, in line with the findings of Benard *et al*.^43^. Notably, while Rotenone is known to mainly mediate its toxic function via inhibiting complex I of mitochondria^17^,^50^, 6-OHDA may induce a direct effect on mitochondria, but its toxic effects appear to be mostly linked to an increase in ROS levels^18^,^19^,^44^.

OS has previously been reported to trigger mitophagy^34^,^35^. Moreover, certain oxidative stimuli were recently described to induce distinct mitochondrial structures that interact with lysosomal compartments independent of mitophagy, comprising “mitochondria-derived vesicles” and “mitochondrial spheroids^36^,^51^”. In this light, mitospheres presented here were also not linked to Parkin-mediated mitophagy. However, our results suggest that mitospheres differ from the described mitochondria-derived vesicles and mitochondrial spheroids due to a) the missing link to the lysosomal compartment, b) the dependence on the fission factor Drp1 and/or c) distinct morphological features, including the size and the engulfment of cytosolic components. Further in-depth studies are needed to unravel the structural and functional differences between these newly described mitochondria-derived structures related to OS. However, these structures observed in cell culture seem to reflect specific functional mitochondrial responses related to different OS-conditions and thus help to understand the role of aSyn in mitochondrial homeostasis.

We found that aSyn substantially prevented the formation of H_2_O_2_- and 6-OHDA-induced mitospheres, suggesting that it modulates the mitochondrial response to OS. Interestingly, mitosphere formation induced by OS was also observed in rat primary cortical neurons from wild type animals showing a robust expression of endogenous aSyn. However, again the additional overexpression of human aSyn considerably reduced the mitochondrial alterations, supporting that a general increase in aSyn levels has a protective effect on mitosphere generation. Previous studies have shown that overexpression of aSyn increases fragmentation of mitochondria^32^,^33^. We also observed an increase in fragmentation of the mitochondrial network in aSyn-overexpressing cells under basal conditions. However, the aSyn-induced fragmentation was profoundly distinct from this induced by OS. In contrast to the OS-related spherically-shaped and hyperpolarized mitospheres, aSyn promoted the formation of short, but not punctate mitochondria, without influencing the MMP. Thus, we suggest that different pathways contribute to aSyn-induced mitochondrial fragmentation and OS-related mitosphere formation – one being triggered by aSyn and one prevented. In the present study, inhibition of Drp1, a key molecule involved in mitochondrial fission, significantly blocked H_2_O_2_-induced mitosphere formation. Accordingly, other studies also demonstrated a role of Drp1 in OS-associated fragmentation of mitochondria^34^,^46^,^49^. In line with the concept that OS-related mitospheres are generated via a different pathway than aSyn-induced mitochondrial fragmentation, it has been reported that the latter occurs independent of the fission factor Drp1^32^,^52^. Our results further suggest that aSyn plays a dichotomic role in the regulation of mitochondrial fusion, mediated via MFN1 a key protein in the fusion of the mitochondrial outer membrane, and OPA1, crucial for the fusion of the inner mitochondrial membrane. In particular, while OS did not influence the two MFN1 bands detected ~85 kDa (representing the 84 kDa MFN1 isoform, frequently shown as a double band^53^,^54^,^55^), it induced a strong reduction in the 60 kDa MFN1 band which was observed as the most prominent band in the mitochondrial fraction (see also Methods section). Moreover, OS led to alterations in different OPA1 isoforms. We detected a reduction in long OPA1 isoforms, paralleled by an increase in short OPA1 isoforms, suggesting an increased OPA1 cleavage, which has been linked to mitochondrial fragmentation^27^,^28^,^29^. Both, the alterations in MFN1 and OPA1 levels may foster the presence of mitospheres by preventing the refusion with the mitochondrial network. aSyn prevented this OS-related reduction of 60 kDa MFN1, and also attenuated the changes in OPA1 levels, suggesting that aSyn inhibits the downregulation of fusion under OS. Notably, overexpression of aSyn *per se* reduced the level of one of the higher molecular weight MFN1 bands (~85 kDa) and this of the 60 kDa MFN1 isoform, indicating that aSyn attenuates fusion of mitochondria under basal conditions. Additionally, aSyn expression itself modulated the levels of OPA1. In total, aSyn reduced OPA1 levels, while increasing the level of a short OPA1 isoform (band 5). Thus, our results support that the aSyn-associated mitochondrial fragmentation under basal conditions is related to a reduced activity of key proteins in the mitochondrial fusion machinery, i.e. MFN1 and OPA1. Future studies are needed to address whether the aSyn-related reduced formation of Drp1-dependent mitospheres is directly linked to this influence of aSyn on mitochondrial fusion. Previous studies suggested a direct effect of aSyn on the fusion^33^ or also fission^32^ of the mitochondrial membrane, independent of classical fission and fusion proteins. However, the aSyn-induced fragmentation has been shown to be, at least partially, compensated by the overexpression or downregulation of fusion or fission proteins, respectively^33^. Furthermore, Guardia-Laguarta *et al*. reported that mutant aSyn increases OPA1 processing^52^. Yet, they did not observe increased mitochondrial fragmentation due to the overexpression of wild type aSyn. Nevertheless, it is important to note that - in contrast to the OS-induced mitochondrial fragmentation in the form of mitospheres - we did not find an association of the wild type aSyn-related mitochondrial fragmentation with alterations in the functionality of mitochondria, as reflected by oxygen consumption, the MMP and ATP levels. Moreover, aSyn overexpression *per se* did also not influence cell viability. This is in line with other studies reporting that the effects of aSyn on mitochondrial morphology appear in the absence of mitochondrial dysfunction^32^,^33^.

Recent studies highlight the role of a disturbance in mitochondrial fission and fusion for PD^56^,^57^,^58^. In particular, it was proposed that the disruption of a proper regulation of Drp1 activity may contribute to PD. Intriguingly, there is also evidence that LRRK2, which is – like aSyn - linked to autosomal dominant PD, interacts and regulates dynamin-related GTPases involved in mitochondrial fission and fusion, including Drp1, Mfn1 and OPA1^59^, indicating potential mechanistic parallels for aSyn and LRRK2.

OS-induced mitospheres display an alteration of the MMP, which usually reflects changes in mitochondrial activity^60^, and mitosphere formation was paralleled by a reduction in oxygen consumption, indicating a decrease in mitochondrial function. In general, mitochondrial dysfunction has been linked to a depolarization of the mitochondrial membrane. Although an increased MMP may mirror an increase in the proton gradient and thus an enhanced mitochondrial respiration, mitochondrial hyperpolarization may also reflect pathological conditions. For instance, an increased proton gradient has been shown to stimulate the generation of ROS^61^,^62^. Thus, hyperpolarized mitospheres may even enforce the H_2_O_2_-induced OS. Moreover, mitochondrial hyperpolarization has been reported as an early event followed by apoptosis^63^,^64^,^65^,^66^,^67^. Accordingly, our results suggest that formation of hyperpolarized mitospheres precedes Casp3 activation. Interestingly, while OS-related mitospheres show an intense MMP, as reflected by the MT staining and validated by flow cytometry using two different MMP-sensitive dyes, several previous studies describe a general depolarization of the MMP upon OS^48^,^49^. Notably, we observed a loss of the MMP-dependent MT staining during apoptosis, suggesting that the initial mitochondrial hyperpolarization is followed by a depolarization. Similarly to our results, Peters *et al*.^68^ found a H_2_O_2_-induced hyperpolarization of the MMP under low concentrations and treatment periods for which changes in cell viability were not detected.

aSyn overexpression not only reduced mitosphere formation, but also prevented Casp3 activation and improved the cellular resistance to OS, confirming the link between mitospheres and apoptosis. Vice versa, this supports that aSyn protects cells by preventing mitosphere formation and thus influences the mitochondrial response to OS.

Remarkably, we only detected a protective effect of aSyn under H_2_O_2_ and 6-OHDA treatment, but not for Rotenone, further supporting the link between the protective effect of aSyn on mitochondrial homeostasis and stress conditions that trigger mitosphere formation in combination with Caspase3 activation.

In line with our results, some studies already suggested a protective role of aSyn in the cellular response to OS^13^,^14^,^69^,^70^, and also revealed an anti-apoptotic function of aSyn^71^. In contrast, elevated aSyn levels, in particular in aggregated and mutant forms, have been shown to enhance basal levels of ROS and/or trigger the cellular susceptibility to OS^72^,^73^,^74^,^75^,^76^. Modification and aggregation of aSyn may lead to a toxic gain of function within the neurodegenerative process. However, our results reveal a protective role of aSyn in mitochondrial homeostasis under OS. Therefore, aSyn mutation, modification and/or aggregation may also directly interfere with this protective function in mitochondrial stress response and thereby contribute to the progression of PD. Intriguingly, both posttranslational modifications and aggregation of aSyn may also be stimulated by sustained OS, which is potentially even enforced in the presence of elevated aSyn levels^77^,^78^, illustrating the dichotomy of the interconnection between aSyn and mitochondrial homeostasis during disease progression.

Notably, while we detected a strong reduction in 6-OHDA-induced Casp3 activation due to aSyn overexpression, 6-OHDA abolished the anti-apoptotic effect of aSyn in a previous study^79^. Interestingly, in this study, the findings have been associated with changes in aSyn aggregation. Also for Rotenone, an increase in aSyn aggregation has been reported in some previous studies^50^,^80^. However, our results do not give evidence for alterations in aSyn aggregation under the conditions applied by us. The discrepancy between different studies concerning the effect of certain stressors on aSyn aggregation, certainly relies on exposure time, concentration, expression level and model systems used. Notably, studies showing intracellular aSyn aggregation under different OS-conditions usually used much longer incubation periods (>24 h or 48 h^50^,^80^,^81^,^82^), suggesting that particularly a longer exposure time is needed in order to induce aSyn aggregation. Yet, since our results suggest that the protective effect of aSyn under certain stress conditions does not - or at least not only - depend on the aggregation status of aSyn, it is likely that the functional impact of aSyn on mitochondrial homeostasis, e.g. on fission and fusion, decides on whether an increased expression of aSyn is beneficial or not under the influence of an individual stressor. Our findings support that the protective effect of aSyn under OS is - at least partially – related to an attenuation of the reduction in mitochondrial oxygen consumption. Interestingly, an increased mitochondrial translocation of aSyn under H_2_O_2_-treatment has been reported^83^, enforcing the assumption that the aSyn effect may be directly mediated via an enhanced interaction with mitochondria. The isolation and analysis of a crude mitochondrial fraction showed a marginal increase in aSyn levels due to H_2_O_2_ in our study, yet without reaching significance. Thus, future studies are needed to decipher the precise mechanism how aSyn mediates the protective effect on mitochondrial function under OS on a molecular level.

In summary, we report that aSyn confers protection in the mitochondrial response to OS. In particular, aSyn modulates mitochondrial fusion and prevents a specific type of OS-associated and Drp1-dependent mitochondrial fragmentation resulting in mitospheres that are characterized by a spherical shape and hyperpolarization. Mitosphere formation was linked to a reduction in mitochondrial function, the activation of Caspase3 and apoptosis. Therefore, the pathological role of aSyn in the interplay between mitochondria and OS, contributing to the neurodegenerative process in PD, may not only be based on a toxic gain of function due to modification and aggregation of aSyn, but also on an impaired protective role of aSyn in the mitochondrial response to OS. Intriguingly, mitosphere formation and also Caspase3 activation was only observed under H_2_O_2_ and 6-OHDA, but not under Rotenone exposure. Since the beneficial effect of increased aSyn levels was also only detected under OS-conditions leading to mitospheres in combination with Caspase3 activation, it is tempting to speculate that aSyn may contribute to a cellular defense mechanism preserving mitochondria against the consequences of increased OS levels, but does not protect against toxins directly inhibiting mitochondrial function.

## Methods

### Cell culture of H4 and LUHMES cells

#### H4 cells

H4 neuroglioma cells of human origin (ATCC, HTB-148) were cultured in Opti-MEM + GlutaMAX (51985-042, Invitrogen) supplemented with 10% fetal calf serum (FCS; 10270-106, Invitrogen) at 37 °C and 5% CO_2_. 3 × 10^4^ H4 cells/cm^2^ were plated 48 h prior to analysis leading to a final confluency of 90%.

#### LUHMES cells

Lund human mesencephalic (LUHMES) cells were a kind gift from M. Leist and D. Scholz, University of Konstanz, Germany. LUHMES cells are conditionally immortalized cells with neuronal features that can be differentiated by shutting-down the myc transgene leading to uniformly post-mitotic neurons within 5 d^84^. LUHMES cells were plated in 50 μg/ml poly-L-ornithine (Sigma-Aldrich) and 1 μg/ml fibronectin (Sigma-Aldrich) coated flasks/plates and cultured in proliferation medium containing advanced DMEM/F12 (Gibco), 2 mM L-glutamine (Gibco), 1% N-2 supplement (Invitrogen) and 40 ng/ml recombinant basic fibroblast growth factor (bFGF, R&D Systems) at 37 °C and 5% CO_2_. For differentiation, 2 × 10^6^ cells were seeded in T75 flasks in proliferation medium. After 48 h proliferation medium was replaced by differentiation medium containing advanced DMEM/F12 (Gibco), 2 mM L-glutamine (Gibco), 1% N-2 supplement (Invitrogen), 1 μg/ml tetracycline (Sigma-Aldrich), 1 mM dibutyryl cAMP (Sigma-Aldrich) and 2 ng/ml glial cell line-derived neurotrophic factor (GDNF, R&D Systems). After additional 48 h, pre-differentiated LUHMES cells were re-seeded at a density of 1.3 × 10^5^ cells/cm^2^ and kept for another 72 h in differentiation medium (5 d differentiation) prior to treatment.

#### Stably transduced H4 and LUHMES cells

Lentiviral transfer vector (pCMV::aSyn-IRES-GFP) and helper plasmids (pVSVG, pMDL and pRev) were used to produce and purify lentiviral particles as previously described^85^,^86^. Transduction was performed in proliferation medium and GFP-positive single cells from H4 and LUHMES cells, as well as non-transduced controls were selected for further clonal expansion.

### Human fibroblasts, iPSC-derived NPCs, and 4 weeks differentiated neural cultures

Fibroblasts from a healthy Caucasian individual with no history of neurologic disease were obtained by dermal punch biopsy of the upper arm, following informed consent and approval from the Institutional Review Board, University Hospital Erlangen, Germany (no. 4120). All methods were performed in accordance with the relevant guidelines and regulations. Fibroblasts were cultured in media containing DMEM (Gibco), 15% FCS (Gibco), 2 mM L-glutamine (Gibco), 100 u/ml, 100 μg/ml P/S (Gibco) and 2 ng/ml FGF-2 (R&D Systems) and plated in 24-well plates at a density of 6,500 cells/cm^2^, 72 h prior to treatment.

Human induced pluripotent stem cells (iPSCs) were reprogrammed from fibroblasts, by retroviral transduction with SOX2, KLF4 and c-MYC, and OCT3/4. They were tested for pluripotency and differentiated into neural precursor cells (NPCs) as previously described^87^. NPCs were expanded in DMEM/F-12, Glutamax media (Thermo Fisher Scientific) supplemented with 1% N2 and 2% B27 supplement (w.o. Vit. A) in the presence of 20 ng/ml FGF2 (Peprotech) and Pen/Strep (1:100). NPCs were seeded on poly-ornithine and laminin-coated coverslips at a density of 1.25 × 10^5^ cells/cm^2^ into 24-well plates, 24 h prior to treatment. For further differentiation, NPCs were seeded at a density of 1.3 × 10^5^ cells/cm^2^ and differentiated in DMEM/F12 supplemented with 1% N2 and 2% B27 supplement (w.o. Vit. A), as well as 20 ng/ml BDNF, 20 ng/ml GDNF, 200 ng/ml ascorbic acid, 500 μg/ml cAMP, and Pen/Strep (1:100) for 4 weeks prior to treatment (differentiated iPSC-derived neural cultures).

### E18 rat primary cortical neuron cultures

WT Sprague–Dawley and BAC SNCA-transgenic (tg) (described in Nuber *et al*.^88^.) rats were housed in stable social groups of 3–4 animals under standard laboratory conditions, with a 12:12 light:dark cycle and free access to food and water, *ad libitum*. For E18 neuronal cultures, rats were paired in homozygosity (wild type or SNCA-tg) for 48 hours, body-weight of the dams was monitored for two consecutive weeks and pregnant females were then sacrificed by decapitation under isoflurane anesthesia after 18 ± 1 days from the estimated mating date. Cultures of embryonic day 18 (E18) rat primary cortical neurons were prepared in accordance to published protocols^89^. Briefly, dissociated primary neurons were plated onto poly-d-lysine-coated 6 well-plates or coverslips in 24 well-plates at a density of 2 × 10^5^ cells/cm^2^. Cells were maintained in Neurobasal medium (Invitrogen), with B27 serum-free supplement (Invitrogen), 0.5 mM L-glutamine, penicillin (100 U/ml) and streptomycin (100 μg/ml). At day *in vitro* 4 (DIV4) 25% and at DIV7 50% of the conditioned medium was replaced by fresh medium. Cells were analyzed at DIV8. All animal procedures conducted were approved by the local Animal Welfare and Ethics committee of Bavaria, Germany and the animal care and use committees of the Friedrich-Alexander-University Erlangen-Nürnberg (TS1_14) and all methods were performed in accordance with the relevant guidelines and regulations.

### Calcium-phosphate transfection

24 h prior to transfection, H4 cells were plated in 24-well plates (1.8 × 10^4 ^cells/cm^2^). Calcium-phosphate transfection of plasmids encoding human wild type aSyn (pcDNA3.1, CMV promoter), GFP (pcDNA3.1, CMV promoter), γSyn (gamma synuclein V5-p(-EGFP)-N3: GFP was replaced by γSyn-V5, CMV promoter) or human Parkin (pcDNA3.1, CMV promoter) was performed as previously described^72^,^90^,^91^. Transfection efficiency was ~ 20–30%. In order to downregulate Drp1 expression, a shRNA against Drp1 (shDrp1, sequence: 5′-GCAATTGAACTGGCTTATATCCGAAGATATAAGCCAGTTCAATTGC-3′) was created by using the BLOCK-iT™ RNAi Designer (ThermoFisher Scientific), as well as a scrambled control shRNA (Sequence: 5′- CCTAAGGTTAAGTCGCCCTCGCCGAAGCGAGGGCGACTT AACCTTAGG-3′) and inserted into an U6 RNAi Entry Vector (Life technologies). For a better discrimination of cells expressing the shRNAs, a GFP expression cassette (CMV-IRES-GFP) has been cloned into the U6 RNAi Entry Vectors (shDrp1-GFP & shScr-GFP) upstream of the shRNA expression cassettes. Cell treatments and analyses were performed 38 h after transient transfection.

### Cell treatment

Stably transduced (aSyn LUHMES) and control LUHMES (Ctr LUHMES) cells, rat E18 cortical neurons, human fibroblasts, iPSC-derived NPCs, as well as iPSC-derived neuronal cultures were cultured as indicated and treated for 4 h with H_2_O_2_ (50–300 μM, Merck). Separate or 1:1 mixed cultures of stably transduced (aSyn H4) and control H4 (Ctr H4) cells were treated with H_2_O_2_ for 10 min up to 40 h (200–500 μM). For single-dose experiments, aSyn and Ctr H4 cells, as well as transiently transfected H4 cells were treated for 4 h with 300 μM H_2_O_2_, a condition that led to a substantial percentage of cells with mitospheres in controls without inducing changes in cell number (see Results). For the Mdivi (Mdivi-1, Sigma-Aldrich, M0199) pretreatment experiments, H4 cells were pretreated with 50 μM Mdivi for 2 h. Afterwards cell culture medium was replaced with fresh culture medium containing 50 μM Mdivi plus 200 or 300 μM H_2_O_2_ and cells were incubated for another 4 h. The treatment of H4 cells with 50 μM CCCP was performed for 10 min up to 40 h. CCCP (50 mM) and Mdivi (50 mM) stock solutions were prepared in ethanol and DMSO, respectively. The stock solutions of CCCP, Mdivi and H_2_O_2_ were freshly diluted in culture medium prior to treatment. Ethanol, DMSO and fresh culture medium served as vehicle controls (VC) for CCCP, Mdivi and H_2_O_2_, respectively. Treatment of H4 cells with Rotenone (0.2–1 μM) and 6-OHDA (50–200 μM) was performed for 4 or 14 h. Rotenone (30 mM) and 6-OHDA (50 mM) stock solutions were prepared in DMSO and in 0.15% ascorbic acid, respectively and freshly diluted in culture medium prior to treatment. DMSO and 0.15% ascorbic acid served as vehicle control (VC) for Rotenone and 6-OHDA, respectively. Depending on the co-staining, mitochondria were stained by MitoTracker Red CMXRos (MT) or MitoTracker Deep Red FM (MTdr). For the MT, MTdr or LysoTracker Red DND-99 (LT) (Life Technologies) staining, cells were incubated for 30 min with 100 nM MT, 200 nM MTdr and/or 75 nM LT in fresh culture medium, subsequent to the treatments.

### Immunocytochemistry

For immunocytochemical analyses (ICC) cells were plated on 13 mm glass coverslips in 24-well plates. Coverslips for LUHMES cells were HCl-pretreated and coated prior to seeding. After washing with Dulbecco’s Phosphate-Buffered Saline (DPBS, w/o Ca, Mg, Sigma-Aldrich), cells were fixed with 4% paraformaldehyde for 15 min. Cells were washed with Tris buffered saline (TBS, pH 7.4) and blocked with fish skin gelatin buffer (FSGB), containing 50 mM Tris/HCl, pH 7.4, 1% BSA, 0.2% fish skin gelatin, and 0.1% Triton-X 100, for 1 h at room temperature (RT). Cells were incubated with primary antibodies overnight at 4 °C. After washing, fluorescence-labeled secondary antibodies were applied for 1 h at RT. Nuclei were stained with 4′6′-diamidino-2-phenylindol (DAPI, Sigma-Aldrich, D8417). Coverslips were mounted by using Prolong Antifade reagent (P36930, Invitrogen). Images were acquired via an Axio Observer inverted fluorescence microscope in combination with an Apotome and an AxioCam MRm camera (Carl Zeiss AG) by using the ZEN software (blue edition, 2012). Objectives: 20x/0.8, 40x/1.3 oil, 63x/1.4 oil.

### Assessment of mitospheres

The ratio of cells with mitospheres was assessed in a systematic, random counting procedure. Images were randomly selected by the DAPI fluorescence-channel. The same settings and exposure times within each independent experiment were used and counting was performed blinded to the GFP and/or synuclein signal. Cells were scored as “positive” for mitospheres, if at least 15 MT-stained mitospheres (for definition of mitospheres see Results section) per cell were detected. The ImageJ Mito-Morphology Macro developed and approved for use by Ruben Dagda^92^ was applied to analyze the average perimeter, the average area and the interconnectivity (mean area/perimeter ratio) of mitospheres formed under H_2_O_2_ treatment as compared to mitochondria under control conditions. Circularity of mitochondria was calculated using the following equation: f_circ_ = 4π × area/perimeter^2^.

### SDS-PAGE and Western blotting

For Western blot (WB) analyses cells were washed with PBS and lysed on ice in radio-immunoprecipitation assay buffer (RIPA): 1% Nonidet P-40 (NP-40; Roche, 11754599001), 0.1% SDS (Applichem, A1112,0100), 0.5% sodium deoxycholate (Sigma-Aldrich, D6750), 50 mM Tris, 150 mM NaCl, 2 mM EDTA, pH 8.0, supplemented with protease/phosphatase inhibitors (Roche). Lysates were centrifuged at 2,000 g and 4 °C for 10 min and post-nuclear supernatants were kept. Protein concentrations were assessed by the BCA assay (Thermo Fisher scientific). 30–50 μg of protein were separated in NuPAGE^®^ 4–12% Bis-Tris Precast Gels (1.0 mm, 12 well, Invitrogen) under reducing conditions and blotted onto polyvinylidene fluoride membranes (Millipore, Immobilon-FL, PVDF, 0.45 μm). Membranes were blocked in 1% BSA in PBS-T (0.1% Tween20 in PBS) for 1 h. Incubation with the primary antibodies was performed overnight at 4 °C. Fluorescence-labeled secondary antibodies were applied after washing, for 1 h at RT. Detection of βactin, β3tubulin or GAPDH served as control and did not show statistically significant differences for any of the performed experiments. Detection of protein carbonylation: The OxyBlot™ Protein Oxidation Detection Kit (Millipore) was used to analyze protein carbonyl groups reflecting OS levels. For this, cell lysates were prepared in RIPA buffer as described before and mixed 1:10 with reducing agent (Invitrogen). Mice were anesthetized with Isofluorane (Sigma-Aldrich, C8383) and flush-perfused transcardially with 0.9% saline (C57BL/6N, 50 weeks of age). All animal procedures conducted were approved by the animal care and use committees of the Friedrich-Alexander-University Erlangen-Nürnberg and the State of Bavaria, Germany and all methods were performed in accordance with the relevant guidelines and regulations. Mouse half brain tissue was homogenized mechanically in homogenization buffer (50 mM Tris, 150 mM NaCl, 2 mM EDTA, pH 8.0, supplemented with protease/phosphatase inhibitors (Roche)) and subsequent sonification for 30 s on ice. Brain tissue homogenate was centrifuged at 800 g for 10 min at 4 °C and supernatant was kept at −80 °C. Tissue homogenate was further lysed in RIPA buffer and mixed 1:10 with reducing agent. Derivatization reaction, SDS-PAGE, blotting and detection was performed according to the manufacturer with the described modifications. 5 μg of protein lysate from cells or tissue were mixed 1:1 with 12% SDS to a final volume of 5 μl. 5 μl of 1x DNPH solution or 1x Derivatization-Control Solution were added and samples were incubated for 15 min at RT. Afterwards, 3.75 μl of Neutralization Solution was added and samples were loaded on a NuPAGE^®^ 4–12% Bis-Tris Precast Gel. After blotting, PVDF membrane (Millipore, Immobilion-P, 0.45 μm) was washed with 2M HCl for 5 min, 2 × 5 min with methanol and 1 × 5 min with PBST. Blocking was performed in 1% BSA in PBS-T for 1 h and the incubation with the primary antibody (rabbit anti-DNP, included in the kit) was performed overnight at 4 °C. Alexa 647 donkey anti-rabbit antibody was used as secondary antibody for 1 h at RT. Ponceau S (Sigma-Aldrich) staining was performed for 5 min and membrane was washed with distilled water until proteins bands were clearly detectable. Fluorescence signals, as well as signals from Ponceau S staining were detected via a Fusion Fx7 detection system (Peqlab GmbH) and analyzed with the software Bio1D version 15.02.

### Preparation of mitochondrial fraction

A crude mitochondrial fraction was prepared via differential centrifugation according to the protocol of Wieckowski *et al*.^93^. In brief, Ctr or aSyn H4 cells were harvested and washed with PBS, and resuspended in 1.8 ml of ice-cold IBcells-1 (225 mM mannitol, 75 mM sucrose, 0.1 mM EGTA and 30 mM Tris-HCl pH 7.4). Cells were homogenized via a glass/Teflon Potter homogenizer and centrifuged twice at 600 g for 5 min at 4 °C. Supernatant was collected and centrifuged at 7,000 g for 10 min at 4 °C to obtain a cytosolic fraction containing lysosomes and microsomes, as well as a mitochondria containing pellet. The pellet was gently resuspended in 2 ml of ice-cold IBcells-2 (225 mM mannitol, 75 mM sucrose and 30 mM Tris-HCl pH 7.4) and mitochondrial suspension was centrifuged at 7,000 g for 10 min at 4 °C. Pellet was again resuspended in 2 ml IBcells-2 and centrifuged at 10,000 g for 10 min at 4 °C. For SDS-PAGE and WB, crude mitochondrial fraction was dissolved in RIPA buffer, 10% 10x RIPA was added to the cytosolic fraction and 30 or 40 μg of protein was loaded for both fractions, as well as total cell lysate.

### Size exclusion chromatographic (SEC) analysis of aSyn

Cells were homogenized in 50 mM Tris pH 7.4 containing 150 mM NaCl, 2 mM EDTA and protease inhibitor cocktail (Roche) by using a Potter dounce homogenizer on ice. Cell lysates containing 60 μg total protein were centrifuged at 100,000 g for 1 h at 4 °C prior to loading onto a Yarra SEC 3000 column (Phenomenex). SEC was performed by using 50 mM Tris/HCl (pH 7.4, with 0.2 M NaCl) as an eluent at a flow rate of 0.5 ml/min and by monitoring the UV absorbance at 280 nm. For the first 10 min, 5 fractions with 1 ml each were collected. After this, one fraction of 0.5 ml per minute was collected. For detection of aSyn, collected fractions were applied to a nitrocellulose membrane placed in a dot blot apparatus (Minifold Dot-Blot System, Schleicher & Schuell). After blotting, membranes were probed by using a mouse anti human-aSyn antibody and a goat anti-mouse HRP antibody (Bio-Rad Laboratories). The proteins were visualized with the Super Signal West Pico Chemiluminescent Substrate (Thermo Scientific). To estimate the size of proteins, Gel Filtration Standard (Bio-Rad Laboratories) containing globular proteins was used.

### Sequential extraction of aSyn (solubility assay)

Aggregation of aSyn was evaluated by its solubility using sequential extraction as described by Tofaris *et al*.^94^ with modifications. Briefly, cells were homogenized in TBS buffer (50 mM Tris/Hcl pH 7.4, 150 mM NaCl) containing proteasome inhibitor cocktail (PI, Roche). 60 μg of protein in 65 μl TBS was used for centrifugation (100,000 g, 4 °C, 1 h). After collecting the supernatant (S1), the pellet was resuspended in TBS containing 1% Triton X 100 followed by another cycle of centrifugation. The resulting supernatant (S2) was collected, and the pellet was dissolved in RIPA buffer (50 mM Tris-HCl, pH 7.4, 175 mM NaCl, 5 mM EDTA, 1% NP-40, and 0.5% sodium deoxycholate) containing 0.1% SDS followed by centrifugation. After removing the supernatant (S3), the pellet (P3) was finally solubilized in 8 M Urea/5% SDS. Always the same volume (65 μl) of the respective buffer was added to solubilize the pellets after each centrifugation step. All centrifugation steps were performed at 100,000 g at 4 °C for 1 h. 19 μl of each fraction were used for SDS-PAGE and WB (see above). Detection of aSyn was performed by using nitrocellulose membranes in combination with mouse anti-aSyn antibodies and fluorescence detection.

### Antibodies

Rat anti-human aSyn (15G7, 1:250, Enzo Life Sciences, ALX-804-258-L001), mouse anti-aSyn (Syn1, 1:500, 610786, BD Biosciences GmbH), rabbit anti-activated Caspase3 (aCasp3, 1:500, 9661, Cell Signaling Technology), goat anti-HSP60 (1:500 ICC, 1:1,000 WB, sc-1052, Santa Cruz Biotechnology), mouse anti-Parkin (1:500, sc-32282, Santa Cruz Biotechnology), mouse anti-GAPDH (1:50,000, MAB374, Millipore), mouse anti-MAP2 (1:500, M1406, Sigma-Aldrich), rabbit anti-TOM20 (1:250 ICC, 1:500 WB, sc-11415, Santa Cruz Biotechnology), mouse anti-β3-tubulin (1:1,000, G7121, Promega GmbH), rabbit anti-βactin (1:2,500, ab8227, abcam), rabbit anti-p62 (1:500, PM045, MBL), rabbit anti-V5 (detection of γSyn, 1:500, ab9116, abcam), rabbit anti-OPA1 (1:750, ab42364, abcam), rabbit anti-Fis1 (1:500, AG-25B-0007V-C100, Biomol), rabbit anti-MFN1 (1:1,500, ABC41, Millipore GmbH). In total, three MFN1 bands were detected by this antibody in the mitochondrial fraction. Using 4–12% Bis-Tris SDS-PAGE under reducing conditions, two bands were separated at ~85 kDa, representing the canonical 84 kDa MFN1 isoform 1 (UniProt: Q8IWA4-1), frequently shown as a double band^53^,^54^,^55^. Additionally, a 60 kDa MFN1 band was observed, which was recently also shown by Morcino *et al*.^95^ and may be identical with the 71 kDa MFN1 isoform 3 (Q8IWA4-3). Smaller bands potentially including the 42 kDa MFN1 isoform 2 (Q8IWA4-2) were not prominent in the crude mitochondrial fraction.

Alexa 488 donkey anti-rabbit (1:1,000, A21206, Invitrogen), Alexa 568 donkey anti-rabbit (1:1,000, A10042, Invitrogen), Alexa 647 donkey anti-rabbit (1:1,000, 711-605-152, Dianova GmbH), Alexa 488 donkey anti-rat (1:1,000, A21208, Invitrogen), Alexa 647 donkey anti-rat (1:1,000, 712-605-153, Dianova GmbH), Alexa 488 donkey anti-goat (1:1,000, A11055, Invitrogen), Cy5 donkey anti-goat (1:1,000, ab6566, abcam), Alexa 488 donkey anti-mouse (1:1,000, A21202, Invitrogen), Alexa 647 donkey anti-mouse (1:1,000, 715-605-151, Dianova GmbH).

### Assessment of cell viability and ATP levels

Cell viability was analyzed by measuring total cell numbers, MTS or MTT assay. Total cell numbers: Cells were lifted with Accutase (PAA) and counted with a CASY^®^ Cell Counter and Analyzer System, Model TT (Roche) or a TC20 automated cell counter (BioRad). MTT assay: Cells were plated and treated in 96-well plates. After replacing the culture medium by fresh medium, 20 μl MTT-reagent (Thiazolyl Blue Tetrazolium Bromide, Sigma-Aldrich, M5655, 5 mg/ml in PBS, pH 7.4) was added and cells were incubated at 37 °C for 2 h. The MTT-containing medium was replaced by 100 μl of a MTT-solubility solution (20% SDS in (1:1) N,N-dimethyl-formamide/water). The plate was incubated for 4 h at 37 °C and absorbance was measured at 550 nm. MTS assay: After treatment, culture medium was replaced by fresh medium and cells were analyzed via MTS viability assay (Promega) according to the manufacturer’s protocol. For the detection of ATP levels bioluminescence signals were detected by using the ViaLight™ Plus Kit (Lonza), according to the manufacturer’s protocol and a VICTOR3 TM Multilabel counter (PerkinElmer).

### High-resolution respirometry

48 h prior to respirometry 2.1 million cells were seeded in a 75 cm^2^ flask. 4 h prior to respirometry cells were treated with 300 μM H_2_O_2_. Subsequently, respirometry of intact cells in an Oxygraph-2k system (Oroboros Instruments) was performed according to Schaefer *et al*.^96^. Shortly, cells were trypsinized and resuspended in their conditioned medium at a final concentration of 1 mio/ml. The Oxygraph-2k system was calibrated to air (gain for oxygen sensor: 4), then cells were added to the two chambers and they were sealed to obtain a closed system. Oxygen consumption was calculated as the negative differentiation of the decreasing oxygen concentration in the chambers. The first steady state level of oxygen consumption displays Routine respiration. Addition of 1.25 μM oligomycin (Olg) blocks complex V of the respiratory system, resulting in Leak respiration. Stepwise release of the proton gradient using the uncoupler FCCP (Carbonyl cyanide-4-(trifluoromethoxy) phenylhydrazone; 0.25 μM FCCP/step) reveals maximum respiration called electron transport system capacity (ETS capacity). Finally, addition of 0.5 μM Rotenone (Rot) and 5 μM Antimycin A (AA) blocks mitochondrial respiration, displaying residual oxygen consumption (ROX). Data were analyzed using DatLab version 5.1.0.20 (Oroboros Instruments). At stable plateaus of oxygen flux, time intervals were drawn, quantifying the mean oxygen flux at the respiratory states, which was corrected for ROX afterwards.

### Flow cytometric analyses

Flow cytometry was performed with the three laser BD FACSCanto II (BD) and BD FACSDiva 6 (BD) analysis and quantification software. The forward and sideward scatter signal was used to determine the population of single cells used for measurements. Detection of LT signal: LT-stained H4 cells were lifted via Accutase (PAA), washed with PBS and analyzed. The mean LT signal per cell was analyzed by measuring 50,000 cells per sample. Detection of the mitochondrial membrane potential (MMP): MMP was investigated via TMRE (Invitrogen) and MitoProbe™ DiIC1 staining (Thermo Fisher Scientific). H4 cells were cultured and treated in 24-well plates (TMRE) and in 6-well plates (DilC1). The stock solution for TMRE (1 mM) was prepared in DMSO. The incubation with TMRE (100 nM) and DilC1 (1:2,000) was performed in fresh culture medium for 20 min at 37 °C. The mean cellular fluorescence intensity of TMRE and DilC1 was analyzed by measuring 20,000 and 50,000 single cells, respectively. For the CCCP control, cells were incubated for 10 min with CCCP (50 μM) and TMRE was directly applied without washing in a final concentration of 100 nM. Competitive survival assay (CSA): aSyn H4 (aSyn-IRES-GFP) and Ctr H4 cells were mixed 1:1 and the experiments were performed in 24-well plates. Cells were lifted and the percentage of aSyn H4 (GFP^+^), representing the relative survival compared to control cells, was detected by measuring 20,000 single cells. Detection of activated Caspase3 (aCasp3)-positive cells: 1:1 mixed cultured aSyn and Ctr H4 cells were lifted and stained via fluorescence-labeled antibodies against aCasp3 (Caspase-3 Alexa Fluor 647, 560626, BD Biosciences GmbH). For the staining, cells were fixed by incubating in a 1:4 dilution of Fixation/Permeabilization Concentrate (eBioscience) in Fixation/Permeabilization Diluent (eBioscience) overnight at 4 °C. Cells were washed with Permeabilization Buffer (eBioscience) and blocked for 15 min by using 10 μl of FcR Blocking Reagent (human, Miltenyi Biotech). The samples were incubated for 1 h at 4 °C with the aCasp3 antibodies (1 μl antibody in 100 μl Permeabilization Buffer per sample). After washing with PBS, GFP and aCasp3 signals were detected via measuring 20,000 single cells per sample. The cut-off fluorescence intensities for defining GFP- and aCasp3-positive cells were set by measuring fixed and permeabilized unstained Ctr H4 cells.

### Electron microscopy

For transmission electron microscopy, H4 cells were seeded and grown on Thermanox plastic cover slips (13 mm) (Thermo Fisher Scientific) in 24-well plates. Cells were treated with 400 μM H_2_O_2_ for 4 h, to obtain a high percentage of cells with mitospheres (90–100%). Cells were fixed with 2.5% glutaraldehyde in 0.1 M phosphate buffer, postfixed in 2% buffered osmium tetroxide, dehydrated in graded alcohol concentrations, and embedded in epoxy resin according to standard protocols. Ultrathin sections were stained with uranyl acetate and lead citrate and examined with a transmission electron microscope (EM 906E; Carl Zeiss NTS GmbH).

### Statistical analyses

Statistical analyses were performed using GraphPad Prism (GraphPad Software). All numeric results are reported as mean + standard error of the mean (SEM) and represent data from a minimum of three independent experiments, unless otherwise stated. Statistics are separately described in the corresponding figure legends. Significant differences are depicted in the figures by graphical representation. p < 0.05 was considered as significant = *. p < 0.01 = **. p < 0.001 = ***.

## Additional Information

**How to cite this article:** Menges, S. *et al*. Alpha-synuclein prevents the formation of spherical mitochondria and apoptosis under oxidative stress. *Sci. Rep.* **7**, 42942; doi: 10.1038/srep42942 (2017).

**Publisher's note:** Springer Nature remains neutral with regard to jurisdictional claims in published maps and institutional affiliations.

## Supplementary Material

Supplementary Material
