# Supplementary material for: Alpha-synuclein prevents the formation of spherical mitochondria and apoptosis under oxidative stress
Source: Sci Rep. 2017 Feb 22;7:42942. doi: 10.1038/srep42942 (PMC5320486; doi:10.1038/srep42942)

Alpha-synuclein prevents the formation of spherical mitochondria and apoptosis under oxidative stress

Stefanie Menges, Georgia Minakaki, Patrick M. Schaefer, Holger Meixner, Iryna Prots, Ursula Schlötzer-Schrehardt, Kristina Friedland, Beate Winner, Tiago F. Outeiro, Konstanze F. Winklhofer, Christine A. F. von Arnim, Wei Xiang, Jürgen Winkler, Jochen Klucken

Corresponding author:

Jochen Klucken, MD,
Department of Molecular Neurology,
University Hospital Erlangen, FAU Erlangen-Nürnberg, 91054, Germany,
E-mail: Jochen.Klucken@uk-erlangen.de

Supplementary Material

Figure S1: Mitospheres are detectable after 4 h of H_2O_2 treatment.

H4 cells were treated with 300 µM H_2O_2 and mitochondria were stained with MT. Changes in mitochondrial morphology are shown in the time frame of 10 min to 4 h after treatment.

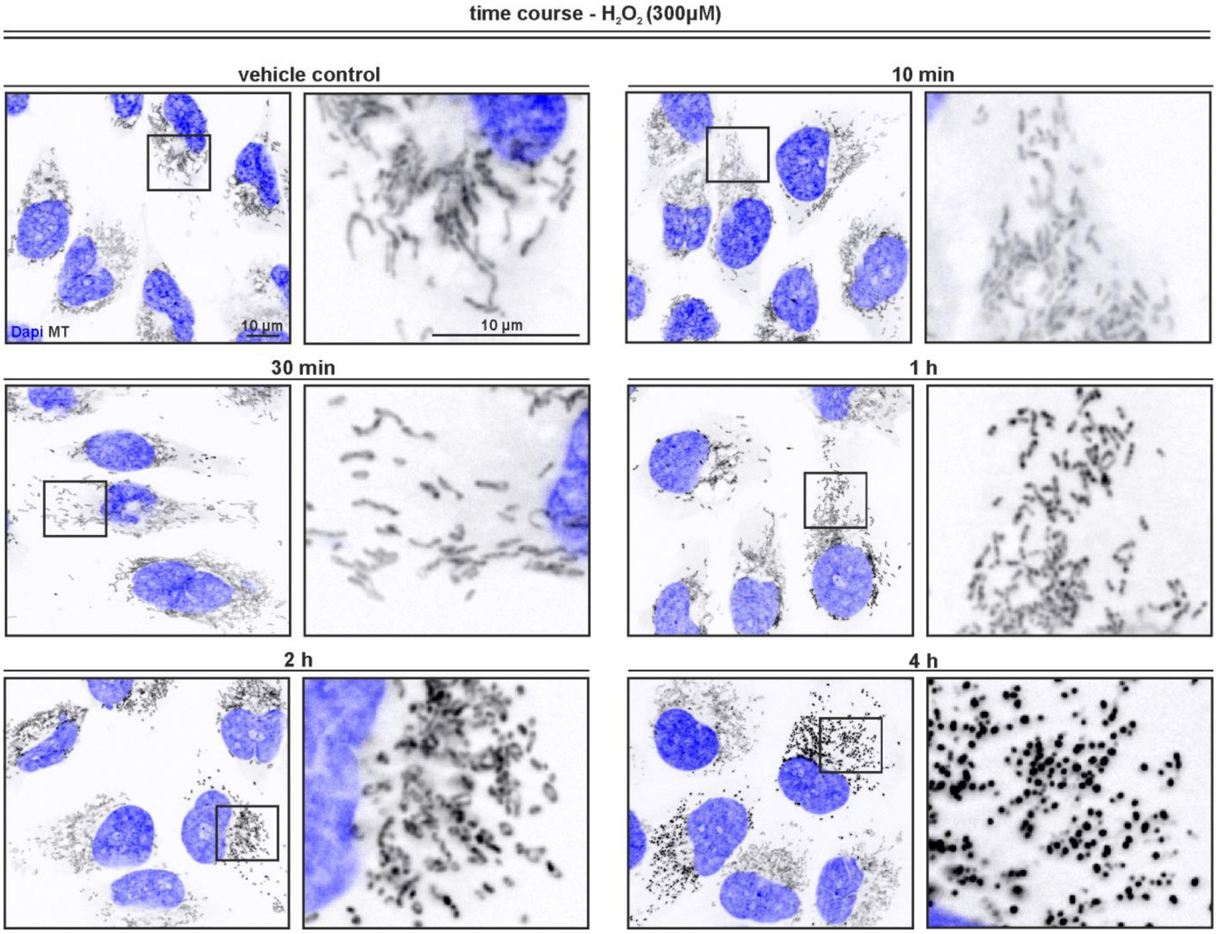

Figure S2: The impact of H_2O_2 treatment on cell number, protein carbonylation and aSyn oligomerization.

(**a**) H4 cells were treated with different concentrations of H_2O_2 for 4 h. Representative light microscopic pictures, as well as the quantification of cell number are depicted. Concentrations up to 400 µM H_2O_2 did not led to significant changes in cell number (One-way ANOVA (repeated measures) Dunnett's Multiple Comparison Test, n=3, p=0.9954 (vehicle control (VC) vs. 200 µM), p=0.9621 (VC vs. 300µM), p=0.8944 (VC vs. 400µM), p=0.0462 (VC vs. 500µM)). (**b**) An increase in protein carbonylation was detected in H4 cells 4 h after the treatment with 300 µM H_2O_2, as assessed via DNP-derivatization and WB. The level of protein carbonylation was in a range comparable to brain tissue lysate of aged mice (C57BL/6N, 50 weeks of age). Membrane depicted on the right side shows the corresponding Ponceau staining. For the quantification of protein carbonyl levels in H_2O_2-treated H4 cells, signal intensity of the sample lanes was quantified within the marked area (unpaired two-tailed t-test, n=3, p=0.0327, area was chosen according to best signal to background ratios) and normalized to the signal obtained via Ponceau S staining. deriv. ctr = derivatization control (H_2O_2-treated H4 cell lysate (lane 7) and mouse brain lysate (lane 10) prepared with Derivatization-Control Solution instead of DNPH), neg. ctr = sample contains DNPH but no protein. WesternC = protein standard. (**c**) Size exclusion chromatography was used to analyze the influence of H_2O_2-treatment (300 µM, 4 h) on aSyn oligomerization in aSyn H4 cells. aSyn signal for the fractions covering the retention times from 0 to 27 min are depicted. For both VC and oxidative stress (OS)-condition the fractions in which aSyn was detected reflected a molecular weight range in which monomeric aSyn is expected [30,31]. Therefore, according to SEC, we did not find an OS-induced increase in aSyn oligomerization under the conditions used. The retention times of the standards Thyroglobin (670 kDa), γ-globulin (158 kDa), Ovalbumin (44 kDa), Myoglobin (17 kDa) and Vitamine B12 (1.35 kDa) are shown. (**d**) The distribution of aSyn in TBS soluble, Triton-X soluble, RIPA soluble, as well as in the insoluble fraction dissolved in Urea / 5% SDS was analyzed via WB for VC-treated cells and cells treated with 300 µM H_2O_2 for 4h and did not give evidence for an alteration of aSyn solubility under OS-condition.

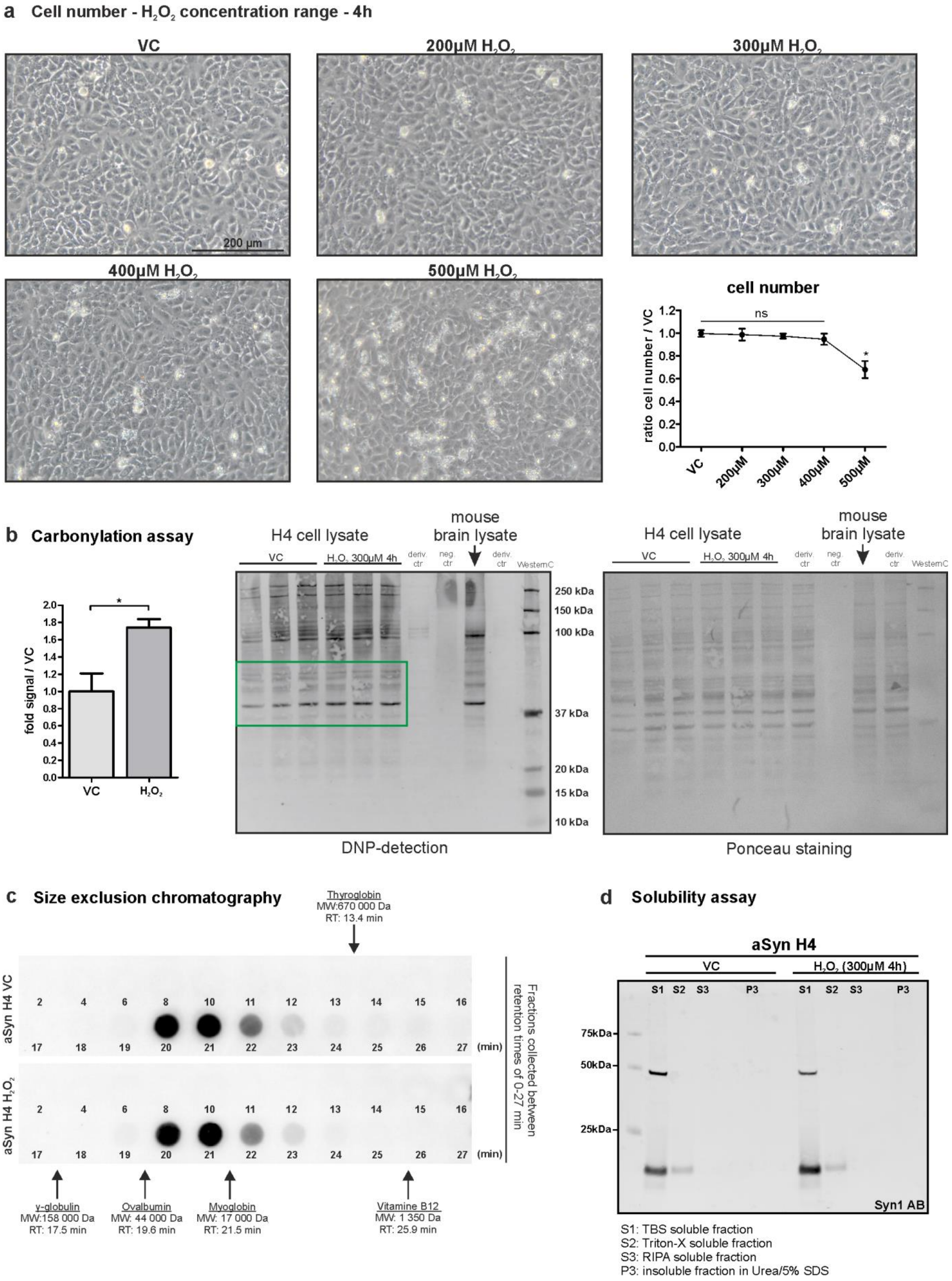

a Cell number - H2O2 concentration range - 4h
VC
200µM H2O2
300µM H2O2
200 µm
400µM H2O2
500µM H2O2
cell number
ratio cell number / VC
ns
*
1.2
1.0
0.8
0.6
0.4
0.2
0.0
VC
200µM
300µM
400µM
500µM
b Carbonylation assay
fold signal / VC
*
2.0
1.8
1.6
1.4
1.2
1.0
0.8
0.6
0.4
0.2
0.0
VC
H2O2
H4 cell lysate
mouse brain lysate
VC
H2O2 300µM 4h
deriv. ctr
neg. ctr
deriv. ctr
WesternC
250 kDa
150 kDa
100 kDa
37 kDa
20 kDa
15 kDa
10 kDa
DNP-detection
H4 cell lysate
mouse brain lysate
VC
H2O2 300µM 4h
deriv. ctr
neg. ctr
deriv. ctr
WesternC
Ponceau staining
c Size exclusion chromatography
Thyroglobin
MW:670 000 Da
RT: 13.4 min
aSyn H4 VC
aSyn H4 H2O2
2 4 6 8 10 11 12 13 14 15 16
17 18 19 20 21 22 23 24 25 26 27 (min)
2 4 6 8 10 11 12 13 14 15 16
17 18 19 20 21 22 23 24 25 26 27 (min)
Fractions collected between retention times of 0-27 min
γ-globulin
MW:158 000 Da
RT: 17.5 min
Ovalbumin
MW: 44 000 Da
RT: 19.6 min
Myoglobin
MW: 17 000 Da
RT: 21.5 min
Vitamine B12
MW: 1 350 Da
RT: 25.9 min
d Solubility assay
aSyn H4
VC
H2O2 (300µM 4h)
S1 S2 S3 P3
S1 S2 S3 P3
75kDa
50kDa
25kDa
Syn1 AB
S1: TBS soluble fraction
S2: Triton-X soluble fraction
S3: RIPA soluble fraction
P3: insoluble fraction in Urea/5% SDS

Figure S3: Western blot showing MFN1 levels under H_2O_2 for Ctr and aSyn H4

Ctr and aSyn H4 cells were treated with 300 µM H_2O_2 for 4h and MFN1 levels were detected by WB. The MFN1 band detected at a molecular weight ~60 kDa shows a substantial reduction under H_2O_2 treatment for Ctr H4 cells, which was not detected for aSyn H4 (see also Figures 3 and 4).

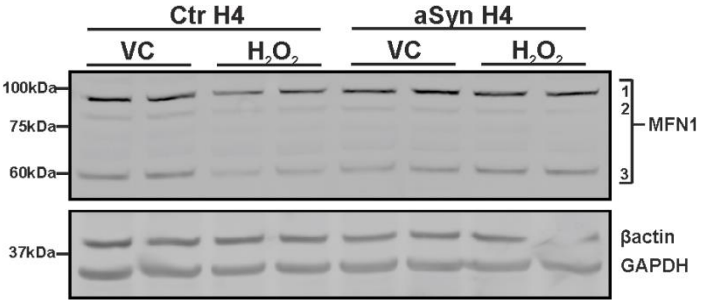

Figure S4: Time course - Parkin-transfected and H_2O_2-treated cells.

(**a** and **b**) H4 cells were transfected with Parkin and treated with 300 µM H_2O_2. After the indicated time points, cells were stained for p62, MT, and Parkin (**a**) or Hsp60, MT, and Parkin (**b**). Substantial co-labeling of H_2O_2-induced MT-positive mitospheres with Parkin or p62 was not detected within the analyzed time frame. Moreover, Parkin did not induce the degradation of the mitochondrial network and mitospheres were still visible 40 h after H_2O_2 treatment. Green dashed lines indicate Parkin-positive cells.

a

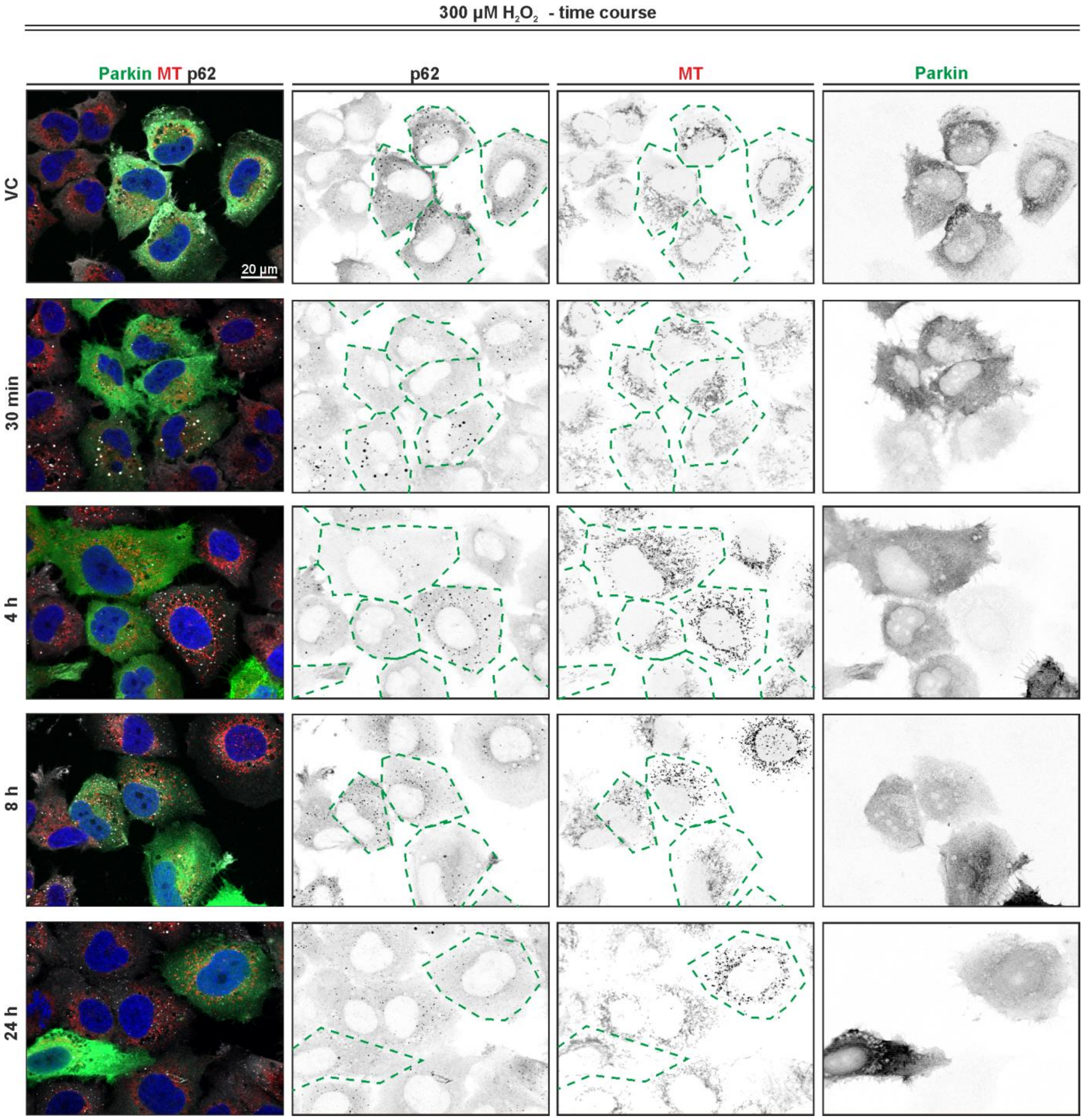

b

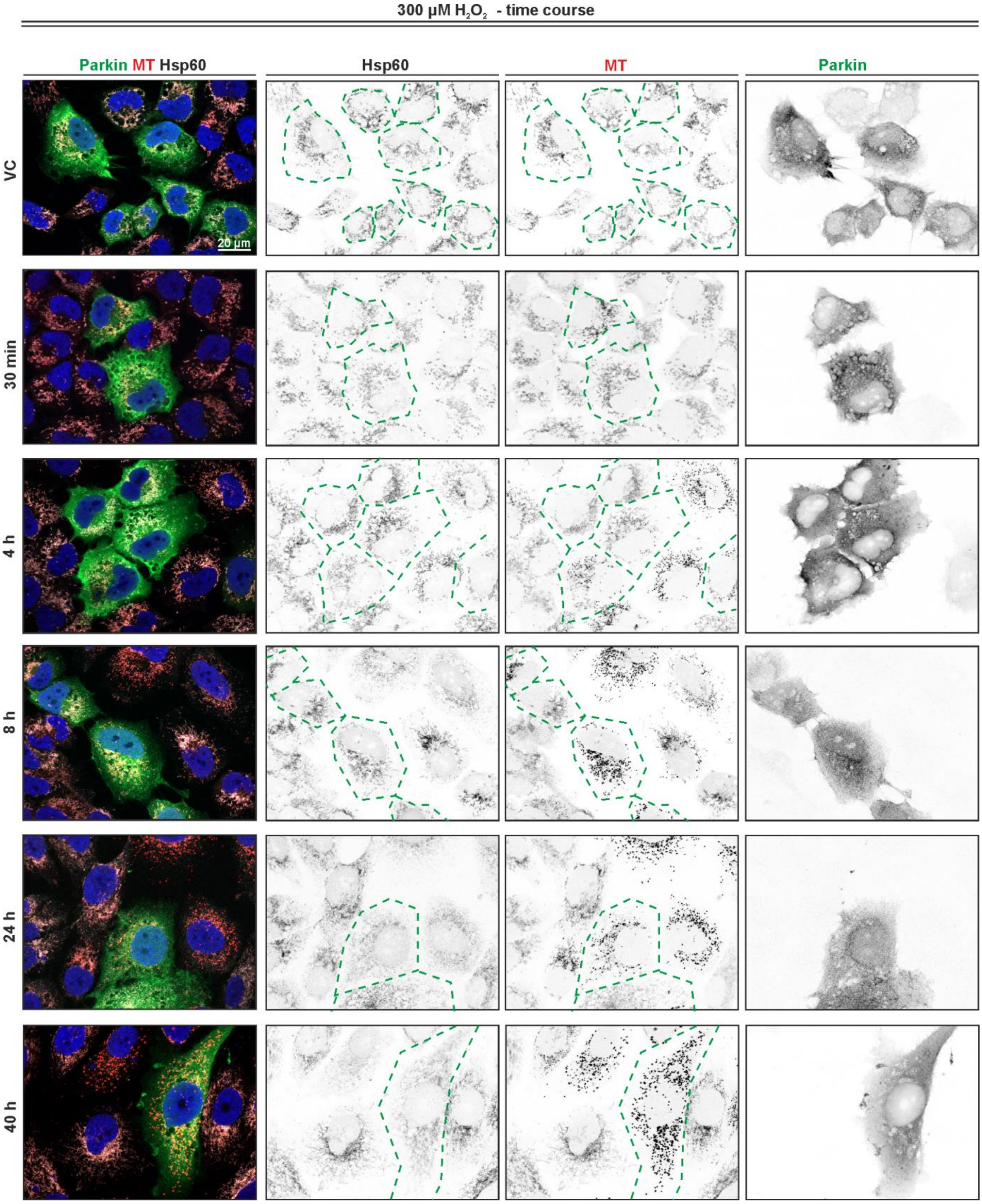
300 µM H2O2 - time course
Parkin MT Hsp60
Hsp60
MT
Parkin
VC
30 min
4 h
8 h
24 h
40 h
20 µm

Figure S5: Time course - Parkin-transfected and CCCP-treated cells.

(**a** and **b**) H4 cells were transfected with Parkin and treated with 50 µM CCCP. After the indicated time points, cells were stained for p62, MT, and Parkin (**a**) or Hsp60, MT, and Parkin (**b**). Co-labeling of mitochondrial structures with Parkin and p62 was detectable after 30 min and 4 h. After 8 h the MT signal, and after 24 h also the Hsp60 signal were lost in Parkin-transfected cells, supporting a complete degradation of the mitochondrial network. Green dashed lines indicate Parkin-positive cells.

a

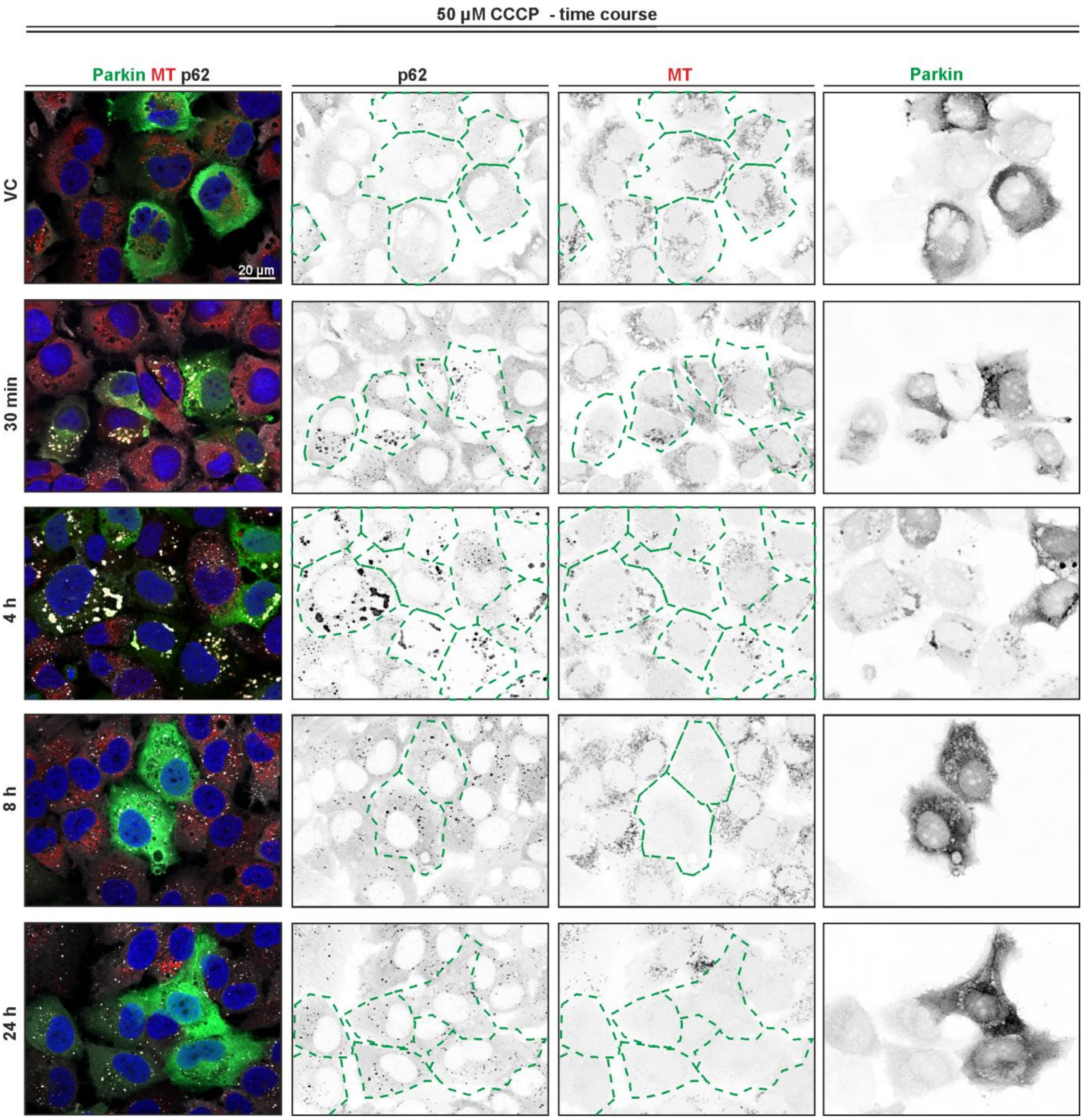

b

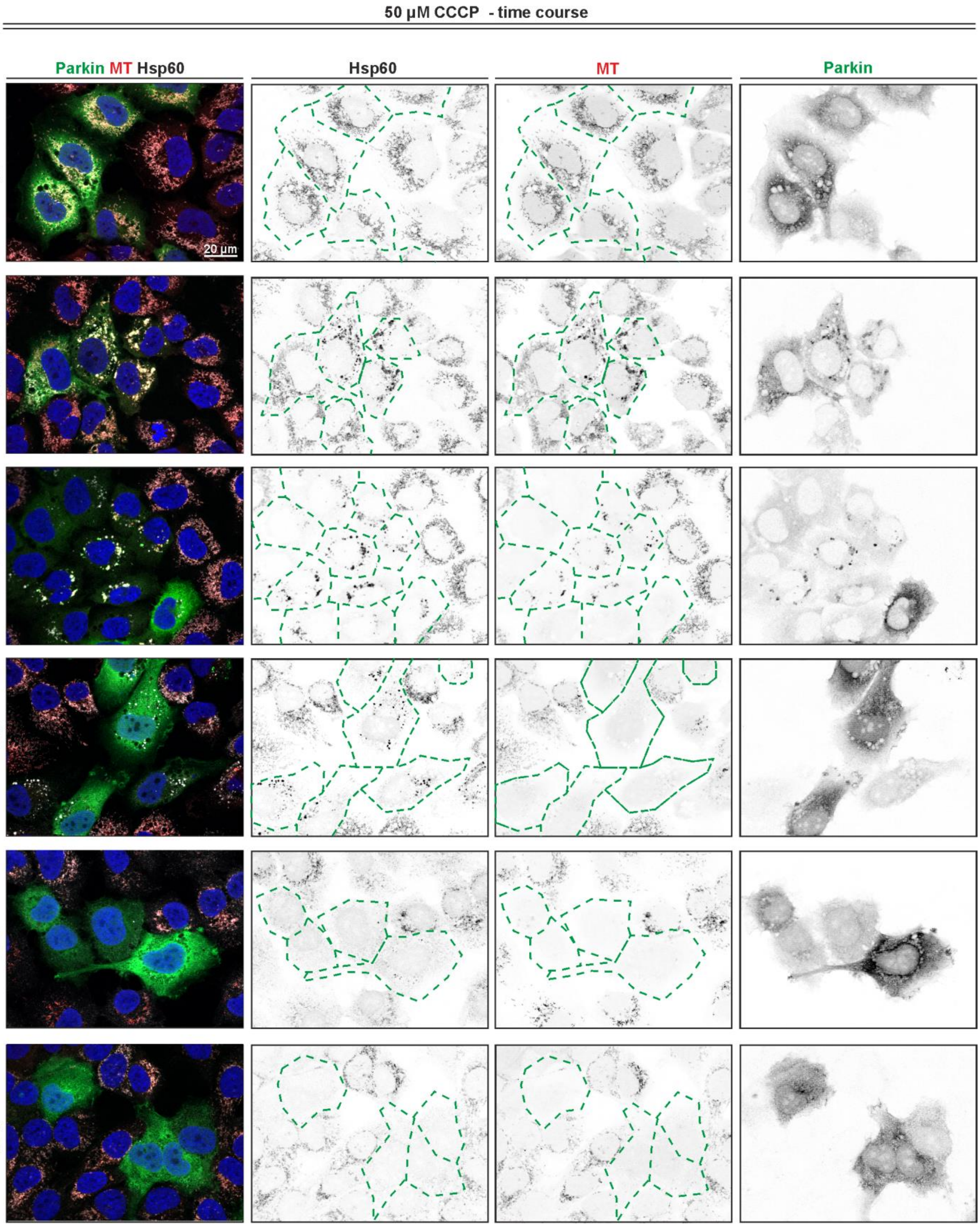
50 µM CCCP - time course
Parkin MT Hsp60
Hsp60
MT
Parkin
VC
30 min
4 h
8 h
24 h
40 h
20 µm

Figure S6: Immunofluorescence staining of aSyn in cells treated with 6-OHDA and Rotenone

Immunocytochemical stainings of aSyn H4 cells treated with 200 µM 6-OHDA and 0.5 µM Rotenone for 14 h do not show an indication for increased aSyn aggregation under these conditions.

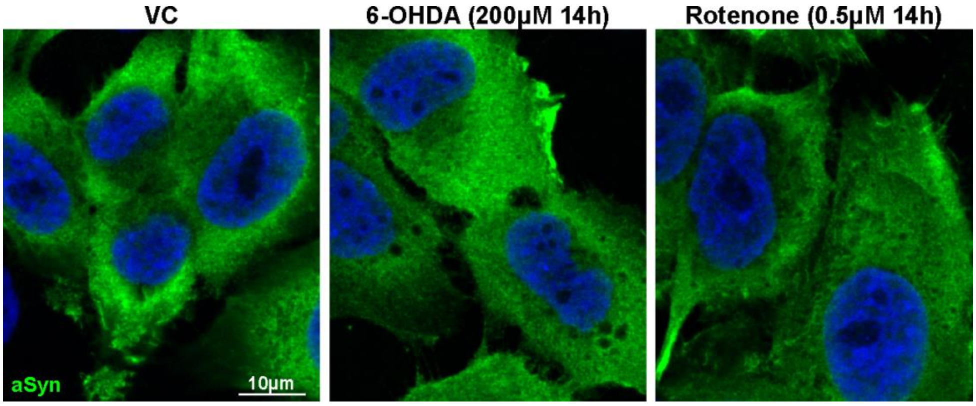

Supplement: Supplementary Material [file srep42942-s1.pdf]
